# A novel GFP-based strategy to quantitate cellular spatial associations in HSV-1 viral pathogenesis

**DOI:** 10.1128/mbio.01454-24

**Published:** 2024-09-09

**Authors:** Deepak Arya, Ujjaldeep Jaggi, Shaohui Wang, Kati Tormanen, Mingtian Che, Simeon Mahov, Ling Jin, Homayon Ghiasi

**Affiliations:** 1 Center for Neurobiology and Vaccine Development, Ophthalmology Research, Department of Surgery, Cedars-Sinai Medical Center, Los Angeles, California, USA; 2 Applied Genomics, Computation, and Translational Core, Cedars-Sinai Medical Center, Los Angeles, California, USA; 3 Department of Biomedical Sciences, Oregon State University, College of Veterinary Medicine, Corvallis, Oregon, USA; The University of North Carolina at Chapel Hill, Chapel Hill, North Carolina, USA; University of Pittsburgh School of Medicine, Pittburgh, Pennsylvania, USA

**Keywords:** ocular infection, virus replication, primary infection, latency, GFP, image mass-cytometry, immune cells

## Abstract

Periodic reactivation of herpes simplex virus type 1 (HSV-1) triggers immune responses that result in corneal scarring (CS), known as herpes stromal keratitis (HSK). Despite considerable research, fully understanding HSK and eliminating it remains challenging due to a lack of comprehensive analysis of HSV-1-infected immune cells in both corneas and trigeminal ganglia (TG). We engineered a recombinant HSV-1 expressing green fluorescent protein (GFP) in the virulent McKrae virus strain that does not require corneal scarification for efficient virus replication (GFP-McKrae). Next-generation sequencing (NGS) analysis, along with *in vitro* and *in vivo* assays, showed that GFP-McKrae virus was similar to WT-McKrae virus. Furthermore, corneal cells infected with GFP-McKrae were quantitatively analyzed using image mass cytometry (IMC). The single-cell reconstruction data generated cellular maps of corneas based on the expression of 25 immune cell markers in GFP-McKrae-infected mice. Corneas from mock control mice showed the presence of T cells and macrophages, whereas corneas from GFP-McKrae-infected mice on days 3 and 5 post-infection (PI) exhibited increased immune cells. Notably, on day 3 PI, increased GFP expression was observed in closely situated clusters of DCs, macrophages, and epithelial cells. By day 5 PI, macrophages and T cells became prominent. Finally, immunostaining methods detected HSV-1 or GFP and gD proteins in latently infected TG. This study presents a valuable strategy for identifying cellular spatial associations in viral pathogenesis and holds promise for future therapeutic applications.

**IMPORTANCE:**

The goal of this study was to establish quantitative approaches to analyze immune cell markers in HSV-1-infected intact corneas and trigeminal ganglia from primary and latently infected mice. This allowed us to define spatial and temporal interactions between specific immune cells and their potential roles in virus replication and latency. To accomplish this important goal, we took advantage of the utility of GFP-McKrae virus as a valuable research tool while also highlighting its potential to uncover previously unrecognized cell types that play pivotal roles in HSV-1 replication and latency. Such insights will pave the way for developing targeted therapeutic approaches to tackle HSV-1 infections more effectively.

## INTRODUCTION

Herpes simplex virus type 1 (HSV-1) infection is widespread and, in the US, over 70% of the adult population has antibodies to HSV (1–3). HSV-1-induced corneal scarring (CS), broadly referred to as HSK, is the leading cause of infectious blindness in developed countries (4), and HSK is much more likely to occur following recurrent HSV infection (5–9). In the US, approximately 500,000 people annually experience recurrent ocular HSV episodes that require doctor visits, medication, and in severe cases, corneal transplants. Despite considerable efforts, neither a vaccine to prevent ocular HSV infection nor safe and effective approaches to eliminate latent HSV infections have been developed. Following ocular infection, the virus travels *via* anterograde and retrograde transport between the site of infection and ganglia of the infected host (10, 11). After establishing latent infection in peripheral sensory neurons of an infected host, the virus may reactivate and travel back to the original site of infection, causing recurrent disease (12–14).

Corneal cells are the site of HSV-1 infection, and the cornea includes epithelial cells, keratocytes, and endothelial cells in three distinct layers (15). The eye is an immune-privileged site, with immune privilege being maintained via mechanisms that attenuate both innate and adaptive immune responses (15, 16). Thus, the immune-privileged status of the eye in general, and the cornea in particular, involves suppressing certain immune cell functions. In contrast to most tissues in the body, the cornea does not normally contain blood or lymphatic vessels (17, 18). However, vascularization of the cornea and T cell infiltration can occur following surgical manipulation or in certain corneal diseases or viral infection. Previous studies showed that healthy rodent and human corneas lack detectable T cells as determined by RT-PCR and immunostaining (15, 19–25), and we recently found infiltration of CD45^+^, CD4^+^, CD8^+^, NK, NKT, B cells, DCs, macrophages, monocytes, and neutrophils as soon as 1 h after corneal infection (26).

Studies from various groups have used different HSV-1 strains, including KOS, RE, F, 17+, McKrae, and many others (27–31). Except for the McKrae strain that is a virulent ocular HSV-1 strain that does not need corneal scarification for efficient ocular virus replication, other HSV-1 viruses require corneal scarification. We previously reported that corneal scarification significantly affects the number and type of infiltrating immune cells even in corneas of uninfected mice, implying that results generated by corneal scarification before HSV-1 infection are likely associated with corneal wounding rather than with infection (26). Here, we asked what cell types are infected during primary infection using a GFP-tagged virus. Therefore, we constructed a recombinant HSV-1 McKrae virus expressing GFP upstream of gD without affecting gD expression. The gD is essential for viral binding to host cell receptors and subsequent entry, with studies demonstrating that mutations or deletions of gD inhibit viral propagation *in vivo* (32). This virus expressed high GFP levels in tissue culture and is similar to its parental virus regarding virus replication in the eye, eye disease, latency, and reactivation. Thus, infection with this virus does not require corneal scarification, and we used it to monitor GFP expression in infected mouse corneas on days 3 and 5 PI and in infected mouse trigeminal ganglia (TG) on days 3, 5, and 30 PI. Using image mass cytometry (IMC), we detected GFP expression along with CD11b, CD11c, F4/80, CD45, CD163, CD3, CD4, CD8α, CD68, Ly-6G, iNOS, FoxP3, and E-cadherin on days 3 and 5 PI. We also directly detected GFP expression in some latent TG by immunohistochemistry (IHC) and RT-PCR and these results were confirmed by gD expression using IHC and RT-PCR.

The important points that emerged from this study are a) GFP was faithfully detected as a surrogate of McKrae virus; b) IMC enabled identification of adjacent GFP-positive immune cell clusters; and c) latent TG showed a subclinical reactivation phenotype, with co-expression of GFP and gD or HSV-1 and GFP. Collectively, our results highlight the potential use of IMC to quantitatively profile immune cell dynamics in murine corneas infected with GFP-McKrae virus. Thus, IMC provides a novel platform with which to study spatial interactions between virus and immune cells. Identification of such dynamic processes will be a critical step in deciphering the contributions of various immune cells to HSK.

## RESULTS

### Construction and characterization of a recombinant virus expressing GFP in HSV-1 strain McKrae (GFP-McKrae)

**Fig 1:**
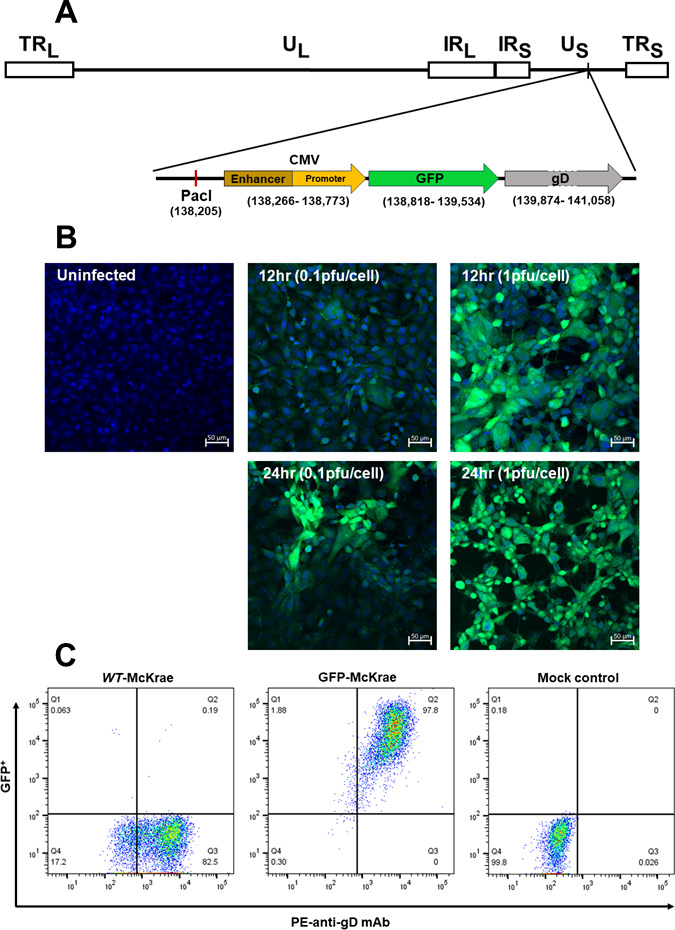
Construction and expression of the GFP-McKrae virus. (**A**) Schematic diagram of GFP-McKrae virus showing HSV-1 GFP-McKrae genome. TR_L_ and IR_L_ represent terminal and internal (or inverted) long repeats, respectively. TR_S_ and IR_S_ represent terminal and internal (or inverted) short repeats, respectively. U_L_ and U_S_ represent long and short unique regions, respectively. gD gene: gray arrow. GFP: Green arrow upstream of gD. One unique PacI site upstream of CMV enhancer/promoter is shown. (**B**) Detection of GFP expression *in vitro*. Subconfluent RS cell monolayers were infected with 0.1 and 1 PFU/cell for 12 and 24 h or mock-infected. Infected cells were isolated and prepared for IHC analysis (see Materials and Methods). Panels show representative infected RS cells. (**C**) FACS analyses of infected RS cells expressing gD and GFP. Subconfluent RS cell monolayers were infected with 0.05 PFU/cell of GFP-McKrae, WT parental McKrae, or mock-infected. At 18 h PI, the cells were harvested, reacted with anti-gD antibody, and FACS analysis was performed (see Materials and Methods).

HSV-1 has previously been modified to express fluorescent reporter genes that distinguish viral infection and pathogenesis in HSV- strains 1 KOS, RE, H25, SC16, and 17 (27–31). For efficient ocular infection, these viruses require corneal scarification that, by itself, stimulates immune cell trafficking into the eye, as we previously reported (26). Among all known HSV-1 strains, McKrae is the only virulent ocular strain that does not require corneal scarification. Thus, to identify infected cell types in the corneas of ocularly infected mice during primary infection and to detect GFP in TG during latency, we constructed a recombinant virus expressing GFP under control of the CMV promoter into the unique short (Us) region between gJ (US5; 137712–138125) and gD (US6; 139762–142585) in HSV-1 strain McKrae (Fig. 1A) (33, 34). The region between gD and gJ is approximately 400 bp and does not code for any gene and does not affect gJ or gD expressions. The presence of GFP in GFP-McKrae virus was verified by PCR and next-generation sequencing (NGS) of the whole viral genome (Gene Bank, accession ID: Mutant Human alpha herpesvirus one clone recombinant GFP-McKrae genomic sequence; PP680711), confirming presence of the GFP gene and one unique PacI site in the virus. In contrast to GFP-McKrae virus, we expected sequencing of the parental McKrae virus (Gene Bank, accession id OL638991.1) to confirm the presence of GFP and two PacI sites.

To determine whether GFP-McKrae virus expresses GFP protein, RS cells were infected with the virus at 0.1 or 1 PFU/cell for 12 or 24 h. Using confocal microscopy, cells infected with 1 PFU of virus expressed more GFP than did those infected with 0.1 PFU, and cells infected for 24 h expressed more GFP than did those infected for 12 h (Fig. 1B). Subsequently, RS cells were infected with 0.05 PFU/cell of GFP-McKrae and McKrae viruses or mock-infected for 24 h and stained with anti-gD antibody as described in the Materials and Methods. In McKrae-infected cells, 82% were gD^+^ and, as expected, no GFP^+^ cells were detected (Fig. 1C, WT-McKrae, right lower quadrant), whereas 98% of GFP-McKrae cells were gD^+^GFP^+^ (Fig. 1C, GFP-McKrae, right upper quadrant). No gD^+^ or GFP^+^ cells were detected in mock-infected controls (Fig. 1C, mock control). Thus, using fluorescence imaging and flow cytometry, our results suggest that GFP-McKrae virus does express GFP protein.

**Fig 2:**
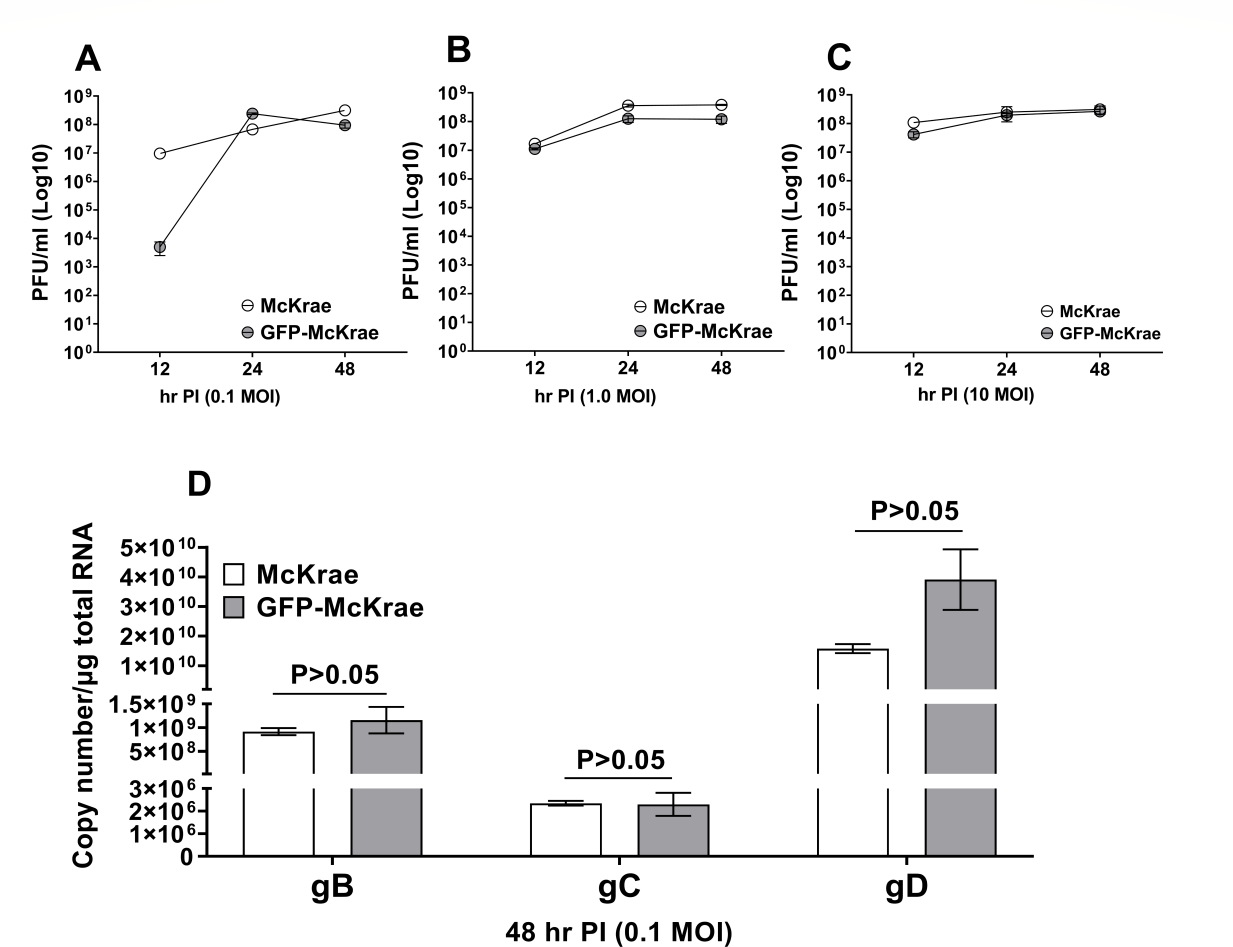
Replication of GFP-McKrae *in vitro*. (**A–C**) Virus replication in RS cells. Subconfluent RS cell monolayers from two separate experiments were infected in triplicate with 0.1 (**A**), 1.0 (**B**) or 10 (**C**) PFU/cell of GFP-McKrae or McKrae for 24 h, as described in the Materials and Methods. Total virus was harvested at indicated times PI by two freeze–thaw cycles. Virus was quantitated by standard plaque assays on RS cells. Each point represents the mean ± SEM (*n* = 6). (**D**) Quantitation of gB, gC, and gD transcripts *in vitro*. Subconfluent RS cell monolayers were infected as described in (**A**). Total RNA was isolated from infected RS cells, and quantitative TaqMan RT-PCR was performed on total RNA as described in the Materials and Methods. Estimated relative copy number of HSV-1 gB, gC, and gD were calculated using standard curves generated for each gene as described previously (35). Each point represents the mean ± SEM (*n* = 6).

To determine how the presence of GFP in GFP-McKrae virus affects virus replication *in vitro*, RS cells were infected with 0.1, 1, or 10 PFU/cell of GFP-McKrae or WT-McKrae viruses for 12, 24, or 48 h. Virus titers in cells infected with GFP-McKrae or WT-McKrae viruses were determined by standard plaque assay and did not show statistically significant differences at any PFU or time point (Fig. 2A through C, *P* > 0.05). These results demonstrate that addition of GFP upstream of gD sequences did not affect virus replication *in vitro*. To determine if the GFP sequence affects expression of other key HSV-1 transcripts, RS cells were infected with GFP-McKrae or WT-McKrae viruses at 0.1 PFU/cell, and the copy number of gB, gC, and gD transcripts were measured by qRT-PCR at 48 h PI (PI). The copy number of gB, gC, and gD in GFP-McKrae-infected cells were similar to those in WT-McKrae-infected cells (Fig. 2D, *P* > 0.05), suggesting that the presence of GFP did not affect levels of gB, gC, and gD expression in infected cells.

**Fig 3:**
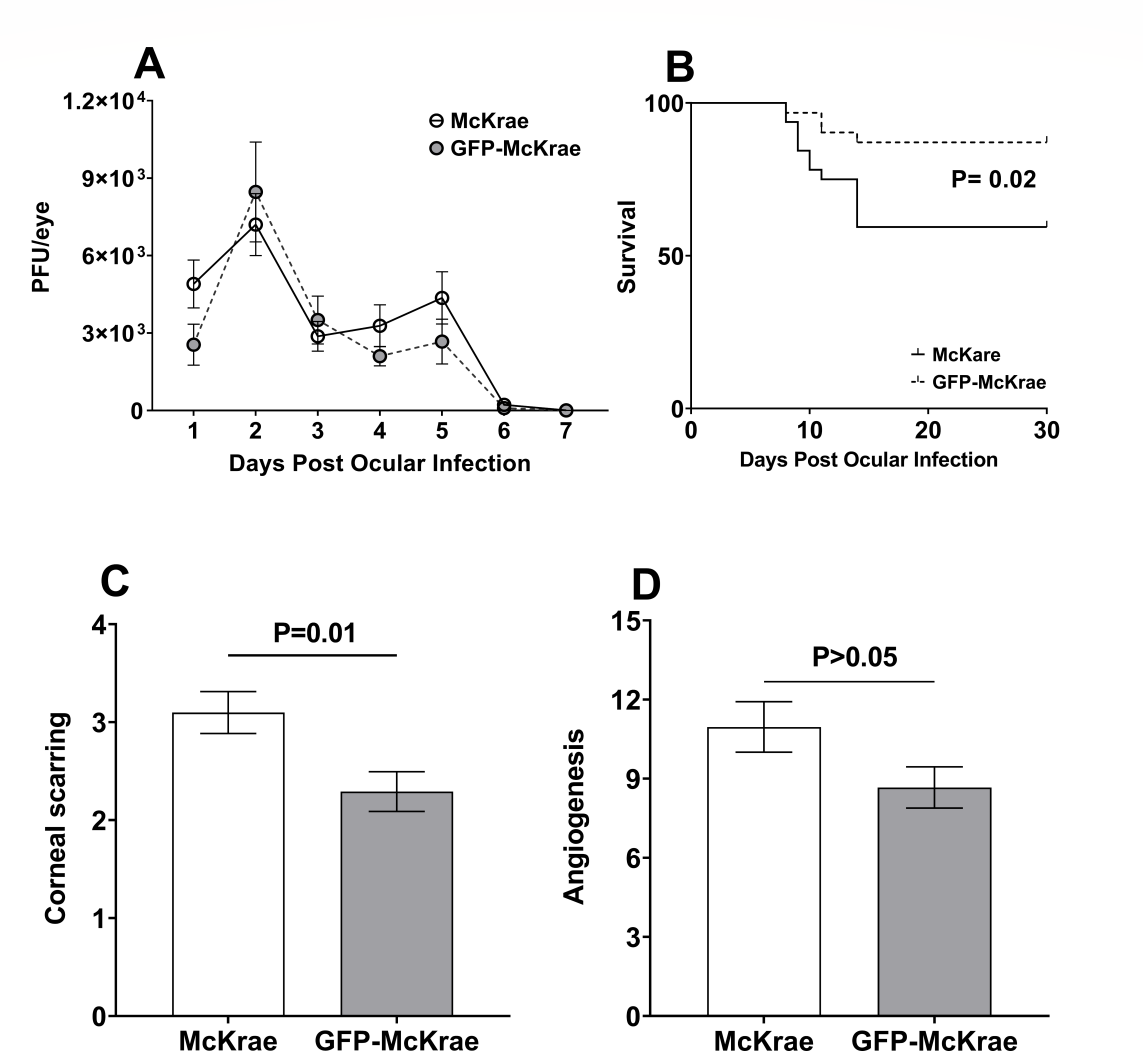
Virus replication in mouse tears, survival, and eye disease in GFP-McKrae-infected mice. (**A**) Virus replication in mouse tears. Female C57BL/6 mice were ocularly infected with 2 × 10^5^ PFU/eye of GFP-McKrae or McKrae virus. Tear films were collected on days 1–7, and virus titers were determined by standard plaque assays. Each point represents the mean titers of 40 eyes from two separate experiments. (**B**) Survival of infected mice was monitored for 30 days PI. The graph represents the average of two independent experiments with 27 of 31 mice surviving in GFP-McKrae group and 19 of 32 surviving in McKrae group (Chi-squared test). (**C and D**) Corneal scarring (CS) and angiogenesis in ocularly infected mice were measured on day 30 PI and represent the average disease from 54 eyes for GFP-McKrae-infected mice and 38 eyes for McKrae-infected mice. Error bars indicate SEM.

The *in vitro* results described above suggested that the presence of GFP did not alter the infectivity of GFP-McKrae virus when compared with WT-McKrae virus. We next asked if the presence of GFP affected virus replication *in vivo*. Mice were ocularly infected with 2 × 10^5^ PFU/eye of GFP-McKrae or HSV-1 McKrae viruses. Virus titers determined in tears of infected mice on days 1–7 PI using standard plaque assays were similar in both groups on all days, suggesting that presence of GFP did not alter virus replication in the eyes of infected mice (Fig. 3A, *P* > 0.05). Survival of ocularly infected mice was recorded for 30 days. In GFP-McKrae-infected mice, 27/31 (87%) survived ocular infection, whereas only 19/32 (59%) of WT-McKrae-infected mice survived ocular infection (Fig. 3B, *P* = 0.02, Chi-squared test), suggesting that GFP had a protective role during ocular infection. CS and angiogenesis in surviving mice were scored in a blinded fashion on day 30 PI as described in the Materials and Methods. A significant reduction in CS was observed between mice infected with GFP-McKrae and WT-McKrae viruses (Fig. 3C, *P* = 0.01), indicating that the GFP sequence impacted eye disease. Conversely, the angiogenesis score showed no significant difference (Fig. 3D, *P* > 0.05). These data suggest that GFP expression driven by the CMV promoter exerts a milder effect on eye disease rather than a broader effect on other related physiological processes such as angiogenesis.

**Fig 4:**
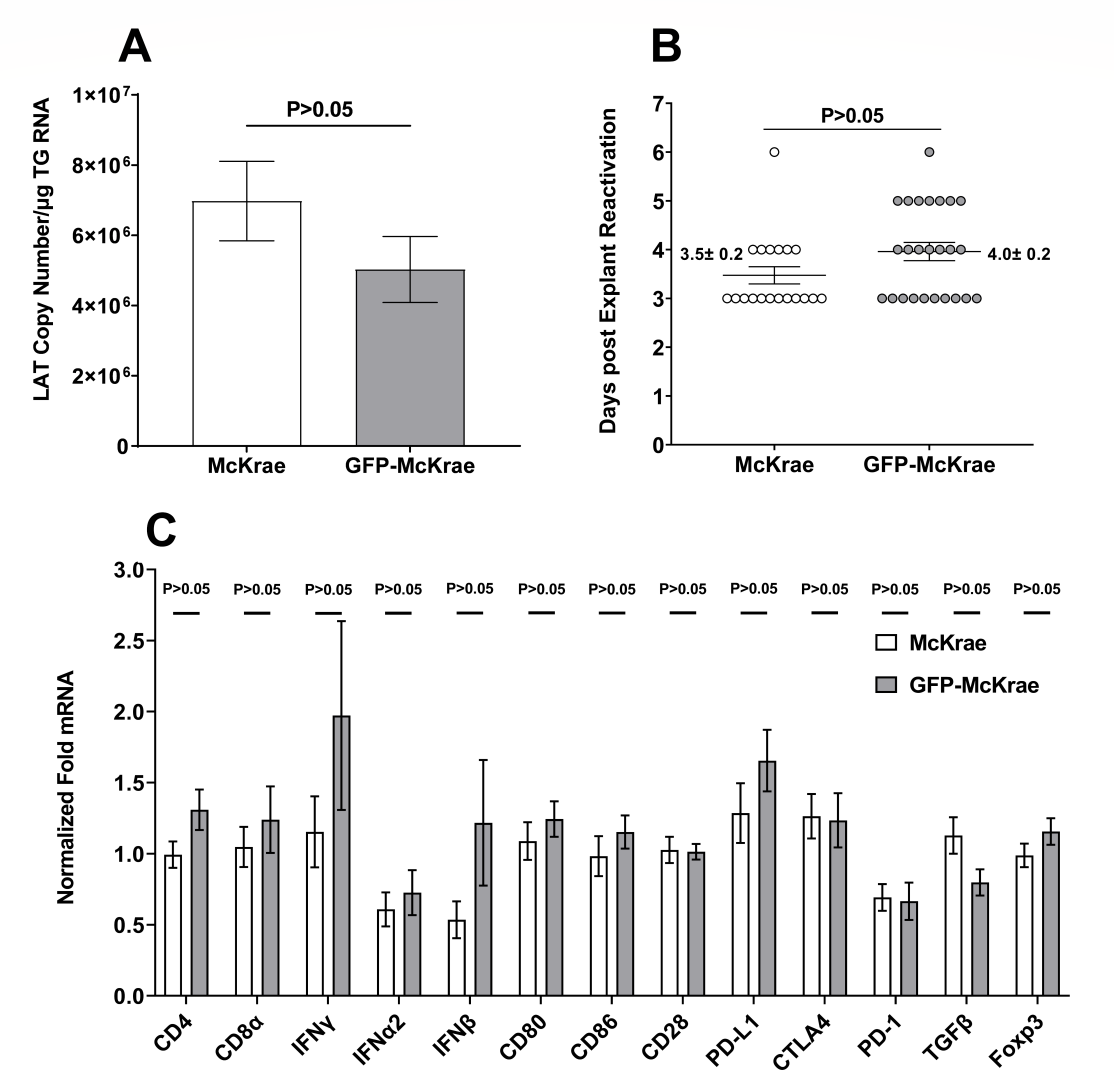
Effect of GFP expression on latency, reactivation, and key host factors in TG of latently infected mice. (**A**) LAT levels in TG of latently infected mice. Female C57BL/6 mice were ocularly infected with 2 × 10^5^ PFU/eye of GFP-McKrae or WT-McKrae. On day 30 PI, TG from latently infected mice were harvested, and qRT-PCR was performed on individual TG from each mouse. Estimated relative LAT copy number was calculated using standard curves generated from pGEM-5317. GAPDH expression was used to normalize relative LAT RNA expression in the TG. Latency is based on 24 TG for GFP-McKrae and 19 TG for McKrae. *P*-values were determined using one-way ANOVA. (**B**) Explant reactivation in TG of latently infected mice. The mice were ocularly infected as above with each virus, and TG from latently infected mice were individually isolated on day 30 PI. Each TG was individually incubated in 1.5-mL of tissue culture media at 37°C. Media aliquots were removed from each culture daily for up to 5 days and plated on RS indicator cells to assess virus reactivation. Results were plotted as the number of TG that reactivated daily. Numbers indicate the average time that TG from each group first showed CPE ± SEM. reactivation is based on 25 TG for GFP-McKrae-infected mice and 19 TG for McKrae-infected mice. *P*-values were determined using one-way ANOVA. (**C**) Effect of GFP on expression of major latency-related host genes in TG of latently infected mice. RNA extracted to measure latency levels (see Fig. 4A) was used to measure expression of CD8, CD4, IFNγ, IFNα2, IFNβ1, CD80, CD86, CD28, PD-L1, CTLA4, PD-1, TGFβ, and FoxP3 transcripts using total RNA as described in the Materials and Methods. Expression of these genes in mice infected with WT-McKrae was used as a baseline control to estimate the relative expression of each transcript in TG of latently infected mice. GAPDH expression was used to normalize expression of each transcript. Each data-point represents the mean ± SEM from 24 TG for GFP-McKrae and 19 TG for McKrae.

To determine the effect of GFP presence on latency and reactivation, mice were ocularly infected with GFP-McKrae or WT-McKrae viruses, and individual TG was harvested on day 30 PI. qRT-PCR of total TG RNA was used to determine latency-associated transcript (LAT) expression levels with no significant difference in LAT expression seen between the two groups of infected mice (Fig. 4A, *P* > 0.05). Time to reactivation was also determined using an *ex vivo* explant assay. Similar numbers of TG reactivated in the two groups (25 of 25 for GFP-McKrae, 19 of 19 TG for McKrae, Fig. 4B). The average time to reactivation between the two groups was also similar, 4.0 ± 0.2 days for GFP-McKrae-infected and 3.5 ± 0.2 days for McKrae-infected mice, suggesting that GFP sequences did not affect latency levels or reactivation in infected mice. The results of analyzing virus replication, eye disease, latency, and reactivation suggested that GFP-McKrae and WT-McKrae viruses were similar. We next asked whether the GFP sequence affected expression of the cellular genes CD8, CD4, IFNγ, IFNα2, IFNβ1, CD80, CD86, CD28, PD-L1, CTLA4, PD-1, TGFβ, and FoxP3 by using qRT-PCR to measure their expression in isolated TG RNA. No significant differences were detected in expression levels of all tested genes (Fig. 4C, *P* > 0.05). Thus, GFP expression did not alter the function of immune genes in TG of latently infected mice. Collectively, these observations showed that GFP expression did not affect the rate or pattern of GFP-McKrae infection, which were similar to those of the parental WT-McKrae virus.

### Spatial profiling using IMC detects spatially adjacent GFP-positive immune cell clusters *in vivo*

**Fig 5:**
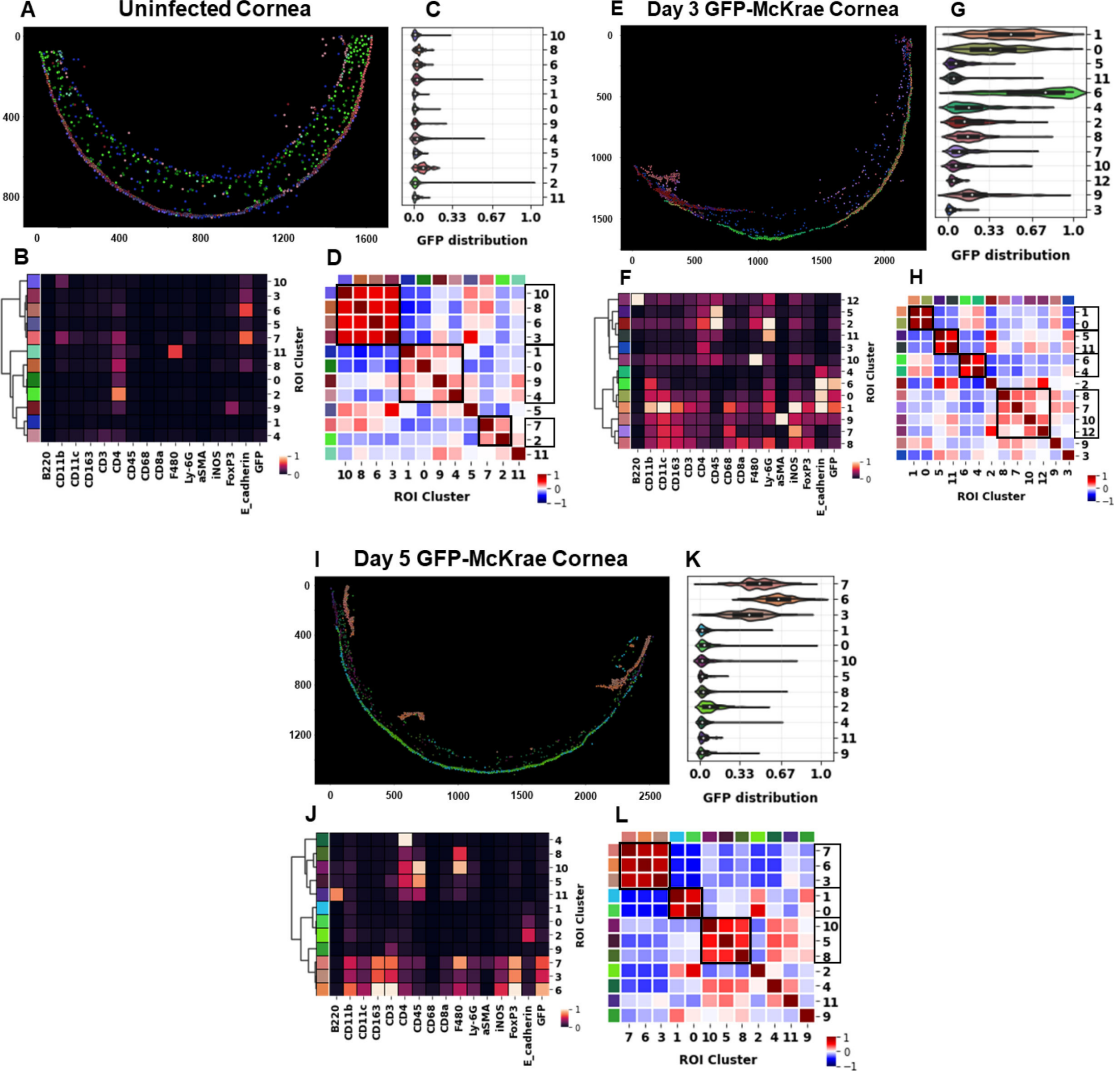
Spatial molecular profiling of mouse corneas infected with GFP-McKrae using IMC. Mice were ocularly infected with 2 × 10^5^ PFU/eye of GFP-McKrae virus as described in Fig. 3 and the Materials and Methods without corneal scarification. Uninfected mice were used as controls. Whole eyes were harvested on days 3 and 5 PI and sectioned. Regions-of-interest (ROIs) were assigned to different corneal regions, and samples were ablated on a Hyperion imaging system (Standard Biotools). Each antibody produced a single image per sample, which was collectively used to construct a multi-image stack in MCD format. Cell segmentation, feature extraction, and normalization were performed. Cell populations were identified using iterative PhenoGraph clustering. Final matrix data were converted to .FCS files within the MATLAB pipeline for clustering and heatmap creation. Panels: (**A–D**) uninfected cornea, (**E–H**) day 3 PI, and (**I–L**) day 5 PI. (A, E, and I): ROIs were combined as single-cell stacked images depicting single-cell map of mouse cornea. (B, F, and J): Heatmaps show expression of structural (aSMA, and E-cadherin), immune cell markers (B220, CD11b, CD11c, CD163, CD3, CD4, CD45, CD68, CD8a, F4/80, Ly-6G, iNOS, and FoxP3), and GFP in assigned cell clusters. Expression was non-log normalized, ranging from 0 to 1. (C, G, and K): Expression/distribution of GFP in assigned cell clusters as a violin plot, ranging from 0 to 1. (D, H, and L): Heatmap of neighbor analysis using HISTOCAT to determine nearest cell neighbor to each start point cell to investigate. These positive, neutral, and negative interactions were then collated to create the overall proportion heatmap for the condition (region, etc.) ranging from 1 (100% of images showed positive interaction) to −1 (100% of images showed negative interaction).

A longitudinal analysis of murine corneas using imaging mass cytometry (IMC) was conducted with customized scripts and standard statistical tools, following the methodology of the Bodenmiller pipeline (36, 37). Two distinct images of day three corneas were analyzed: one of the entire cornea, and another of a small region used for standardization. Single sections of both uninfected corneas and corneas at day 5 PI were similarly analyzed. As IMC has been used successfully to study the spatial arrangement of cells in virus infections (38, 39), we used an IMC-based approach to study whole corneas from uninfected mice, as well as corneas isolated from mice infected with GFP-McKrae virus on days 3 and 5 PI (Fig. 5). Single-cell stacked images of uninfected corneas showed the spatial arrangement of immune cells in the epithelium (Fig. 5A). These cells were arranged in 11 clusters based on expression of immune and non-immune cell markers. Clusters 2, 6, 7, and 11 expressing CD4, E-cadherin and F4/80 showed the presence of CD4^+^ T cells, F4/80^+^ macrophages, CD11b^+^ population of macrophages, neutrophils, and DCs on E-cadherin^+^ epithelium (Fig. 5A). Other markers showed very low or no expression (Fig. 5B). As expected, no GFP expression was detected in any clusters in uninfected mouse corneas (Fig. 5C), confirming that uninfected corneas were not exposed to GFP-McKrae virus, and there was no background GFP staining. An interaction map was created to show the proximity of clusters in the cornea. Notably, clusters 3, 6, 8, and 10 were closely associated (Fig. 5D) and expressed CD11b, CD4, FoxP3, and E-cadherin, suggesting that macrophages (CD11b^+^, F4/80^+^), DCs (C11b^+^), and T cells (CD4^+^, FoxP3^+^) were adjacent in uninfected corneal epithelia (E-cadherin^+^). We also identified spatially associated clusters 0, 1, 4, and 9, composed of T cells that expressed CD4, FoxP3, and CD3. The presence of T cells, macrophages, and DCs in uninfected corneas is consistent with our previous reports (26, 40).

On day 3 PI with GFP-McKrae virus, the single-cell map showed a marked increase in immune cell clusters 0–12, (Fig. 5E and F). Markers expressed at highest levels on day 3 included B220, CD11b, CD11c, CD45, F4/80, Ly-6G, aSMA, iNOS, E-cadherin, and GFP. Markers expressed at moderate levels included CD163, CD4, CD68, CD8a, and FoxP3. Cluster 12 included B cells (B220^+^); cluster one included M1 macrophages (CD11b^+^, iNOS^+^, CD68^+^), cluster nine included myofibroblasts (aSMA^+^), clusters 2 and 11 included neutrophils (CD11b^+^, cluster 1 Ly6G^+^); clusters 1 and 8 included T cells (CD3^+^, CD4^+^, FoxP3^+^), and clusters 4, 6, 0, and 1 included epithelial cells (E-cadherin^+^). GFP distribution was measured in each cluster, with the highest GFP expression in cluster 6, and formed epithelial cells, which confirms higher virus infectivity (Fig. 5G). Epithelial cells, macrophages, and DCs from clusters 1 and 0 also had high GFP expression. Clusters 2, 4, 8, and 9, including epithelial cells, neutrophils, fibroblasts, and DCs (CD11b^+^, CD11c^+^), expressed lower levels of GFP. Neighborhood analysis showed that epithelial cells, macrophages, and DCs, with high GFP expression, were adjacently situated (Fig. 5H). Macrophages and B cells were also closely situated. These findings were confirmed by examining a different sample where single ROI was analyzed (Fig. S1). The combined data suggest that infection of mouse corneas with GFP-McKrae virus caused an influx of immune cells on day PI, which formed distinct, close clusters containing epithelial cells and GFP-McKrae virus.

On day 5 PI, the single-cell cornea map showed immune cells together with corneal cells (Fig. 5I). Markers expressed at high levels on day 5 included B220, CD163, CD3, CD4, CD45, F4/80, FoxP3, and GFP (Fig. 5J); and those expressed at moderate levels included CD11b, iNOS, E-cadherin, and CD11c. Clusters 2–8, 10, and 11 expressed these markers. GFP expression was reduced on day 5 and limited to clusters 3, 6, and 7 (Fig. 5K). These clusters included closely associated T-regulatory cells (CD3^+^, CD4^+^, FoxP3^+^) and M2 macrophages (CD11b^+^, F4/80^+^, CD163^+^) (Fig. 5L). Interestingly, the epithelial cells from clusters 0 and 2 did not express GFP, suggesting that virus was cleared from the corneal epithelium. T cells, B cells, and macrophages were also detected on day 5.

The above results indicate that pro-inflammatory immune cells, such as neutrophils, DCs, and M1 macrophages, play important roles in clearing the virus on day 3 PI. The proportion of immune cells increases in anti-inflammatory M2 macrophages, and T-regulatory cells on day 5 PI, which are important to establish memory of the virus infection. The imaging mass cytometry (IMC) has effectively provided the spatial signatures of immune cells on various days post-infection. However, unresolved questions remain regarding the virus–host microenvironment. GFP expression in immune cells indicates two potential scenarios: (a) the immune cells were directly infected by the virus and maintained GFP expression, or (b) the immune cells phagocytosed the infected cells and subsequently acquired GFP expression. Taken together, the IMC-based method provides an improved platform to detect and study immune cell dynamics in uninfected and GFP-tagged HSV-1-infected mouse corneas.

### Expression of GFP-McKrae and McKrae viruses during primary and latent infection *in vivo*

**Fig 6:**
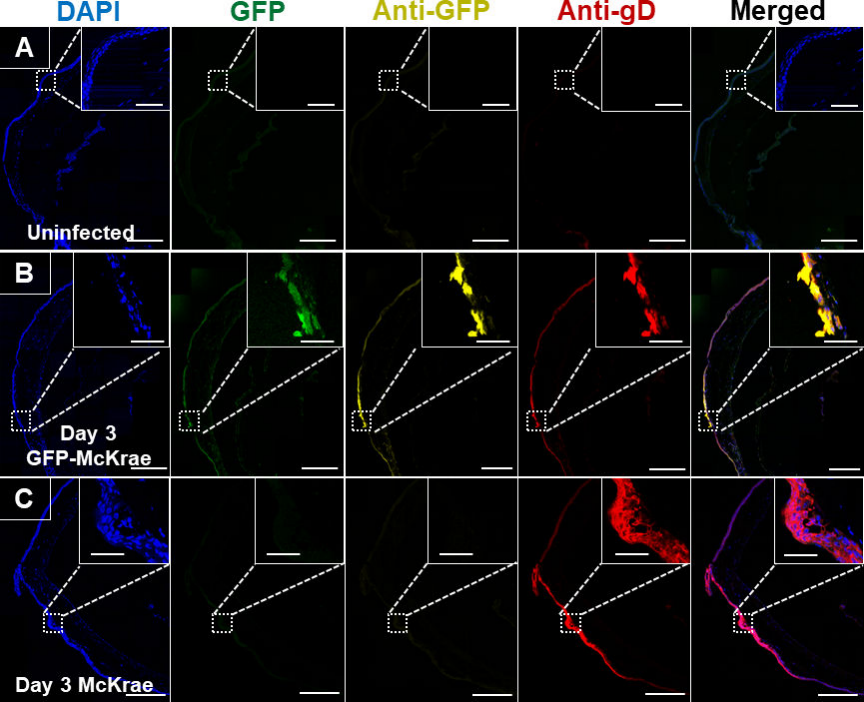
Detection of GFP-McKrae or McKrae virus in mouse corneas on day 3 PI. Mice (three mice per group) were ocularly infected as described above, with and without corneal scarification. Uninfected mice were used as controls. Whole eyes harvested on day 3 were sectioned. Tissue harvesting, sectioning, staining, image acquisition, and analysis were performed as described in the Materials and Methods. Slides were stained with anti-Alexa Fluor 594 (yellow; to detect GFP), anti-Alexa Fluor 647 (red; to detect gD), and DAPI nuclear stain (blue). Endogenous GFP signal was captured at 510 nM. Images were acquired on a 63× immersion oil objective using a Leica confocal microscope. Colocalization was visualized as orange–brown in the merged images. Scalebar represents 250 µM. Enlarged colocalized region insets shown on top right: scalebar represents 50 µM. Panels: (**A**) Uninfected control. (**B**) GFP-McKrae virus. (**C**) McKrae virus.

To examine GFP and gD protein expression in mouse corneas and TG during primary and latent infection, the mice were infected with GFP-McKrae or McKrae virus as described in the Materials and Methods. Eyes and TG were harvested on days 3, 5, and 30 PI, and tissues were sectioned and stained for GFP and gD proteins. Endogenous GFP signal was detected at 510 nM. Corneas were first immuno-stained to check for GFP and gD expression. Uninfected mice, used as controls, were processed in the same way as infected group. No GFP or gD expression was detected in uninfected corneas (Fig. 6A). On day 3 PI, corneal images from mice infected with GFP-McKrae, had lesions with high GFP (endogenous GFP and anti-GFP) and gD expression (Fig. 6B) as well as increased GFP and gD colocalization, suggesting that the virus has similar GFP and gD expression *in vivo*. Corneas from mice infected with McKrae virus had only gD+ lesions (Fig. 6C), with no GFP expression. We next examined GFP and gD expression in the eyes on day 5 PI with GFP-McKrae virus and, as expected, infected corneas had fewer regions with colocalized GFP and gD expression (Fig. S3A), with reduced expression levels compared with expression on day 3. The expression of GFP and gD was observed in patches within the corneal stroma near conjunctiva, whereas the epithelial layer exhibited diffuse and low expression. McKrae virus-infected corneas also expressed gD, but not GFP, as expected (Fig. S3B).

**Fig 7:**
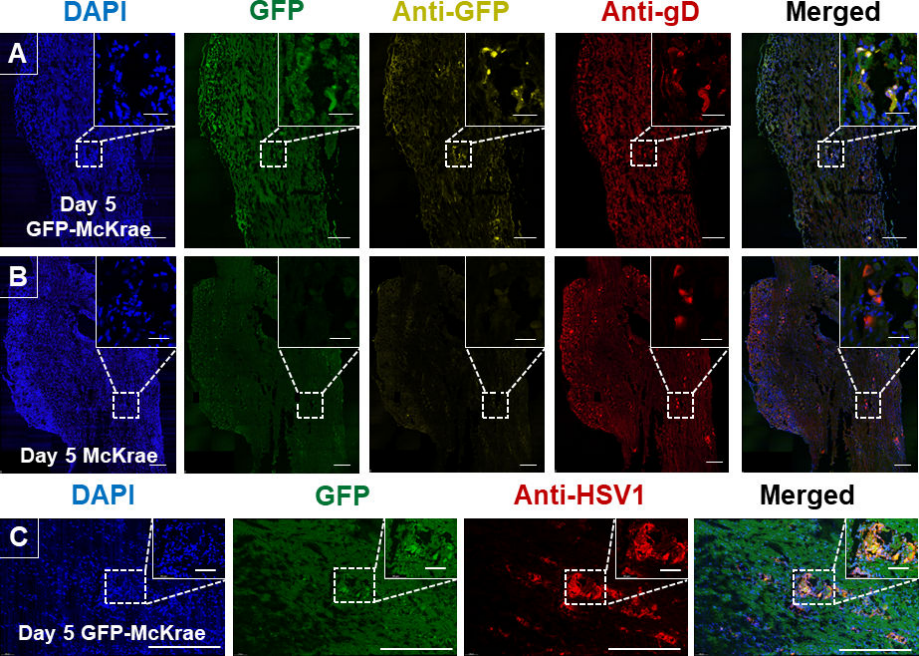
Detection of GFP-McKrae or McKrae virus in mice TG on day 5 PI. Mice (three mice per group) were ocularly infected as described above without corneal scarification. Uninfected mice were used as controls. TG were harvested on day 5 and sectioned. TG staining, image acquisition, and processing were performed as described in Fig. 6. Representative confocal images show GFP staining (detected at 510 nM), anti-GFP (detected with anti-Alexa Fluor 594), anti-gD (detected with Anti-Alexa fluor 647), and nuclear stain (detected with DAPI). Scalebar represents 250 µM. Enlarged colocalized region insets shown on top right: scalebar represents 50 µM. Panels: (**A**) Uninfected control. (**B**) GFP-McKrae virus. (**C**) McKrae virus.

We next examined GFP and gD expression in TG sections. Uninfected TG did not express GFP or gD (Fig. S4A), whereas on day 3 PI with GFP-McKrae virus, confocal images of TG were positive for GFP and gD expression (Fig. S4B), with colocalized expression. On day 3 PI, McKrae-infected TG showed gD-positive regions (Fig. S4C). Strikingly, on day 5 PI with GFP-McKrae virus, multiple TG regions were positive for GFP and gD expression (Fig. 7A), with higher colocalization of GFP and gD. On day 5 PI, McKrae-infected TG continued to have multiple gD-positive regions (Fig. 7B). To confirm our detection of gD expression in infected TG, TG were stained with anti-HSV-1 antibody. On day 5 PI with GFP-McKrae virus, multiple regions were positive for GFP and HSV-1, with colocalization of GFP and HSV1, suggesting a similar expression pattern for both viruses (Fig. 7C). These results suggest that GFP insertion does not interfere with infection or with virus migration to sensory neurons of the TG. Taken together, these results demonstrate that GFP insertion into the virus does not affect virus establishment in the TG or gD expression.

**Fig 8:**
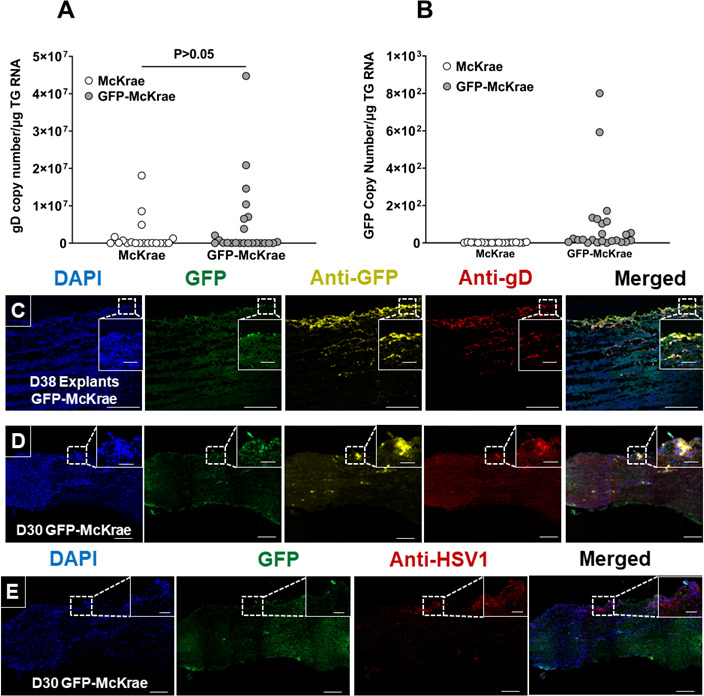
Expression of GFP and gD in latently infected TG on day 30 PI. (**A**) Expression of gD in TG of latently infected mice. Mice were ocularly infected with 2 × 10^5^ PFU/eye of GFP-McKrae or McKrae virus. On day 30 PI, TG from latently infected mice were harvested, and qRT-PCR was performed on individual TG from each mouse. Estimated relative gD copy number was calculated using standard curves generated from pGem-gD1 (43). GAPDH expression was used to normalize relative gD mRNA expression in TG. Expression was based on two independent experiments, with 24 TG from GFP-McKrae and 19 from McKrae viruses. *P*-values were determined using one-way ANOVA. (**B**) GFP expression in TG of latently infected mice. GFP copy number was determined as described in Fig. 8A. The estimated copy number was calculated using standard curves generated from pUC57-GFP-gD plasmid. GFP expression was normalized to endogenous control GAPDH and plotted as mean ± SEM for TG from GFP-McKrae and McKrae viruses. (**C and D**) Expression of GFP and gD in TG of latently infected mice. The mice were ocularly infected with GFP-McKrae virus as described above, and explant TG were used as positive controls (Panel C) for intact TG (panel D). Three mice (six TG) per group were used in the experiment. TG staining was performed as described in the Materials and Methods. Endogenous GFP was detected at 510 nM for all samples. Scalebar represents 250 µM. Enlarged colocalized region insets shown on top right: scalebar represents 50 µM. (**E**) Expression of GFP and HSV-1 in latent TG. TG harvesting, staining, image acquisition, and analysis was performed as described in the Materials and Methods. Briefly, serial sections from latent TG infected with GFP-McKrae virus on day 30 PI were stained with anti-HSV-1 antibody detected with anti-Alexa Fluor 594.

To explore the effect of GFP on latency, the mice were infected with GFP-McKrae or McKrae viruses as described above. RNA isolated from TG of infected mice on day 30 PI were analyzed for gD, and GFP expression by qRT-PCR. Copy numbers were normalized to an endogenous GAPDH control. qRT-PCR analysis showed gD expression in 7 of 24 TG from GFP-McKrae-infected mice and in 3 of 19 TG from McKrae-infected mice, with no significant difference in gD expression between the two viruses (Fig. 8A, *P* > 0.05). The gD expression in TG of McKrae-infected mice varied from 0.5 × 10^7^ to 2 × 10^7^ copies and from 0.4 × 10^7^ to 4 × 10^7^ copies in GFP-McKrae-infected mice. The GFP copy number in latent TG of GFP-McKrae-infected mice (Fig. 8B) was marginally increased in 7 of 24 TG, ranging from 100 to 800 copies in these mice. To determine whether viral proteins could be detected in latent TG sections from GFP-McKrae-infected mice, we examined sections for GFP, gD, or HSV-1. Reactivated explant TG were used to validate the positive GFP and gD signals. The explants displayed widespread GFP and gD expression in different regions of the TG (Fig. 8C). Among GFP-McKrae-infected latent TG, one TG was positive for GFP and gD expression (Fig. 8D), which we carefully examined to determine if the signal was truly positive for the virus. Serial sections were stained with HSV-1 antibody that detected whole virus (Fig. 8E), and the region that was positive for GFP and gD expression (Fig. 8D) was also positive for HSV-1, demonstrating that GFP-McKrae virus was maintained in a latent state in the TG. These results confirm and extend our previous studies that detected both gD RNA and gD protein in TG of latently infected mice (41, 42).

## DISCUSSION

Despite numerous published reports on alpha herpesviruses over the decades using virological, genetic, biochemical, and microscopic approaches, these traditional methods typically lack sufficient spatiotemporal resolution to fully discern the kinetics and dynamics of viral processes. Advancing GFP-based techniques fill this gap, making it possible to understand viral processes by imaging over a time course. Various groups have used recombinant viruses that express fluorescent reporter genes to identify mechanisms and cell types involved in viral pathogenesis, which has revolutionized our understanding of biological processes as never before. Recombinant viruses expressing fluorescent reporter genes could be valuable cloning vectors for subsequent genetic manipulation of the virus that will facilitate efforts to localize live infected cells and tissues without disrupting the cells (44). Indeed, the GFP gene is a very effective tool used in most retrovirus- and herpes virus vector-mediated gene transfer strategies both *in vitro* and *in vivo* (45). In the HSV-1 field, reporter genes are typically incorporated into various HSV-1 strains tailored to the study of different infection or disease stages (27–29, 31, 46–50). However, a significant challenge of such engineered systems exists in their requirement for corneal scarification of the eyes to achieve efficient virus inoculation and propagation, which leaves the HSV-1 field lacking such an important GFP tool. GFP-McKrae enabled real-time visualization and tracking of viral infection and replication within host cells. This modification did not impact replication efficiency, host immune response, or latency establishment. Latency-reactivation assays conducted 30 days PI revealed no significant differences between the recombinant and parental viruses, consistent with previous reports on fluorescent reporter tagging and latency phenotypes (51). We speculate that the observed differences in corneal scarring (CS) and survival may be associated with gD expression under the CMV promoter, necessitating further examination of gD in this context.

Previous studies have demonstrated that mechanical damage to the ocular surface can induce an inflammatory response and subsequent wound healing processes (26, 52). Therefore, HSV-1 studies could be significantly improved by circumventing the need for corneal scarification. To address this need, we constructed a GFP-tagged HSV-1 McKrae virus, which is a virulent ocular isolate that does not require corneal scarification for efficient ocular viral replication and latency establishment. This GFP-based approach facilitates our ability to monitor efficient virus infection while minimizing potential confounding factors associated with tissue damage, thus providing a more accurate understanding of HSV-1 infection in mouse corneas and TG during primary infection and latency-reactivation. Recently, techniques, such as IMC and multiplexed ion beam imaging technology (MIBI), have been used for proteomic analysis of infected tissues. These technologies allow examination of up to 40 different protein markers within a small region. The objective of our current study was to document infiltration of immune cells into infected tissues and determine whether HSV-1-positive cells are uniformly distributed across the whole tissue. To achieve these goals, we used a combination of IMC and a GFP-tagged HSV-1 variant to detect immune cells and explore their spatial associations with the HSV-1 infection. Past studies provide a valuable descriptive analysis of immune cells, pathways, and their functions in managing HSK, but infection status at cell and tissue levels during early stages of virus replication has not been addressed. The interplay between different immune cells in infected tissues plays a critical role in maintaining homeostasis, yet existing barriers to detecting and monitoring these cells during HSV-1 have left us struggling to fully understand the process (53). HSV-1 infection induces a huge influx of innate immune cells, including neutrophils, DCs, gamma delta T cells, and macrophages, causing viral clearance from the cornea and leading to latency (26). The infiltration of immune cells induced solely by GFP is unlikely to produce a broader and more robust effect compared with the infection caused by HSV-1 tagged with GFP. There is a crucial need to understand these cell functions and interactions as they are the primary influencers of HSV-1-associated pathogenesis. We have previously shown the effect of corneal scarification on immune cell infiltrates (26), with initial macrophage infiltration, followed by T cell infiltration at later times PI, demonstrating the importance of targeting macrophages rather than other immune cell types for therapeutic treatment of HSV-1 (26).

Evaluating the role of GFP-tagged HSV-1 (GFP-McKrae virus) *in vitro* and *in vivo* from primary and latent infections showed no major differences in virological or pathological behaviors compared with its parental McKrae virus, but we detected 25 cellular markers able to identify myofibroblasts, plasma cells, epithelium, B cells, T cells, macrophages, DCs, and neutrophils. We examined corneas infected with GFP-McKrae to quantitatively score immune cell types and their spatial association with virus. Our assay revealed the highest GFP expression in epithelial cells, macrophages (CD11b^+^; iNOS^+^; CD163^+^; CD68^+^), DCs (CD11c^+^), B cells, neutrophils, and myofibroblasts (aSMA^+^) on day 3 PI. CD4^+^ T cells and F4/80^+^ macrophages present throughout the stromal region expressed minimal GFP. GFP expression was localized to the cornea on day 3 PI based on IMC and immunostaining results. Interestingly, most immune cells were found at the corneal periphery with few cells at or near the GFP^+^ corneal region, suggesting that macrophages, B cells, and DCs infiltrate into the cornea and stroma through the limbal region. By collecting similar data on day 5 PI, we found a marked shift in GFP expression within corneal immune cells, with expression exclusively in macrophages (CD11b^+^, CD163^+^, F4/80^+^, and iNOS^+^) and T cells (CD3^+^ and FoxP3^+^). These cells also formed spatially adjacent clusters at the corneal periphery. GFP-negative CD4^+^ T cells were scattered throughout the stromal region. Additionally, B220^+^ B cells were detected on days 3 and 5 post-infection (PI), consistently lacking GFP expression. This indicates a possible cell type-specific expression pattern or regulatory mechanism preventing GFP expression in B220^+^ B cells. These findings align with previous reports that identified immune cells at the site of infection (26, 54). The presence of CD4^+^ T cells on day 5 PI marks the beginning of the HSK adaptive immune phase, justifying the presence of T cells associated with disease pathogenesis. On day 5 post-infection with HSV-1, anti-inflammatory M2 macrophages and T-regulatory (Treg) cells play pivotal roles in modulating the immune response. M2 macrophages exert anti-inflammatory effects by secreting cytokines, such as IL-10 and TGF-β, which aid in limiting excessive inflammation and promoting tissue repair. Concurrently, Treg cells contribute to immune regulation by suppressing the activity of effector T cells and maintaining immune tolerance through the production of anti-inflammatory cytokines. Together, these cells help to balance the immune response, reduce tissue damage, and facilitate healing while still addressing the viral infection.

We also used GFP-McKrae virus to investigate the latency phenotype. Previously, viral genes or antigens were thought to be absent during latency. However, development of more sensitive molecular tools has enabled detection of smaller amounts of viral gene products in a few latently infected mouse neurons (55–57). For example, ICP0 and ICP4 expressions have been detected in latent human TG (58, 59). Expanding on this knowledge, we examined GFP and gD expressions in TG of latently infected mice and detected GFP and gD antigens in a few infected mouse neurons, which was validated by staining with HSV-1 antibody to confirm the presence of whole virus. The presence of the HSV-1 signal in the same region confirmed that a small fraction of TG exhibited subclinical reactivation *in vivo*, a phenomenon previously reported by others (55), and we previously detected gD in latently infected TG (41, 42), suggesting either low level expression of viral antigens in a small number of neurons during latency, or that subclinical or abortive reactivations occurs in mice at very low levels.

In summary, for the first time we have shown the utility of using a GFP-based HSV-1 vector to understand complex cellular interactions during primary and latent stages of HSV-1 infection. We expect the results of this work to profoundly impact clinical settings where virulent virions could be easily traced, to help understand viral trafficking, thereby alleviating symptoms of infection. Virions with packaged GFP have been safe and effective tools to address complicated infection models because GFP does not require additional gene products or substrates and GFP does not seem to interfere with cell growth or inhibition (28, 60). By combining GFP tools with IMC-based spatial analysis, we gained a more comprehensive understanding of cellular states relevant to HSV-1 expression that will advance our ability to monitor and improve prevention of recurrent disease.

## MATERIALS AND METHODS

### Construction of GFP-McKrae virus

Using WT-McKrae as the parental virus, we synthesized a plasmid containing the HSV-1 McKrae region from nucleotides 136441 to 139321 together with the complete GFP sequence under cytomegalovirus (CMV) promoter control, which was flanked by two unique Pac1 sequences (GenScript, Piscataway, NJ). This construct was inserted into the Pac1 site of pUC57. The resulting plasmid was designated pUC57-GFP-McKrae. GFP-McKrae recombinant virus was generated by homologous recombination as we previously described (35, 61, 62). Briefly, pUC57-GFP-McK was co-transfected with infectious McKrae DNA using the calcium phosphate method. Viruses from the co-transfection were plated, and isolated plaques were picked and screened for GFP expression. Selected plaques containing GFP gene expression were plaque purified five times and reanalyzed by restriction digestion, immunostaining, and complete genome sequencing (Gene Bank, accession ID: Mutant Human alphaherpesvirus one clone recombinant GFP-McKrae genomic sequence; PP680711).

### Kinetics of viral replication in tissue culture

Rabbit skin (RS) cell monolayers at 70%–80% confluency were infected with 0.1, 1, and 10 PFU/cell of WT HSV-1 strain McKrae, or GFP-McKrae. Virus was harvested at 12, 24, or 48 h PI by subjecting cell monolayers to two freeze–thaw cycles. Virus titers were determined using standard plaque assays on RS cells as described previously (63).

### RNA extraction from infected RS cells

RS cell monolayers at 70%–80% confluency were infected with 0.1 PFU/cell of McKrae or GFP-McKrae virus for 48 h PI. Total RNA was isolated, and RT-PCR was performed using gB, gC, and gD primers. Levels of gB, gC, and gD RNA from infected RS cells were determined using custom gB, gC, and gD primer and probe set as follows: 1) gB-specific primers: forward 5′-AACGCGACGCACATCAAG-3′, reverse 5′-CTGGTACGCGATCAGAAAGC-3′, and probe 5′-FAM-CAGCCGCAGTACTACC-3′; 2) gC (ABI ARCE4XE – amplicon length = 122 bp); and 3) gD-specific primers: forward 5′-GCGGCTCGTGAAGATAAACG-3′, reverse, 5′-CTCGGTGCTCCAGGATAAACTG-3′, and probe 5′-FAM-CTGGACGGAGATTACA-3′. GAPDH was used to normalize viral RNA. Relative copy numbers were calculated using standard curves generated using plasmids pAc-gB1 DNA (for gB) (64), pAc-gC (for gC) (65), and pAc-gD (for gD) (43).

### Cells, viruses, and mice

RS cells were used to prepare virus stocks, culture mouse tear swabs, and determine viral growth kinetics. RS cells were grown in Eagle’s minimal essential medium supplemented with 5% fetal bovine serum. Triple plaque-purified virulent HSV-1 strain McKrae and GFP-McKrae viruses were used in this study. Six- to seven-week-old female C57BL/6 mice (Jackson Laboratory, Bra Harbor, ME) were used.

### Ocular infection

Mice were infected via the ocular route with 2 × 10^5^ PFU of each virus suspended in 2 µL of tissue culture medium (supplemented with 5% serum). Viruses were administered as an eye drop after corneal scarification.

### Virus titration in tears of infected mice

Tear films were collected from both eyes of each ocularly infected mouse on days 1–7 PI using a Dacron-tipped swab (66). Each swab was placed in 0.5 mL of tissue culture medium, squeezed, and the virus titer was determined using a standard plaque assay on RS cells.

### *In vitro* explant reactivation assay

Mice were sacrificed at 30 days PI and individual TG were removed and cultured in tissue culture media as we previously described (67). Briefly, a 100-µL aliquot was taken daily from each culture and used to infect RS cell monolayers. Infected RS cells were monitored daily over 5 days for the appearance of cytopathic effect (CPE) to determine the time at which reactivated virus from each TG first appeared. Because media from explanted TG cultures were plated daily, the time at which reactivated virus first appeared in explanted TG cultures could be determined.

### Immunofluorescence

RS cell monolayers grown on Lab-Tex chamber slides were infected with 0.1 or 1 PFU/cell of GFP-McKrae virus for 12 or 24 h. To examine GFP expression, unfixed and unpermeabilized slides were washed thrice with phosphate-buffered saline (PBS), air dried, fixed with acetone, mounted with Prolong Gold DAPI mounting medium (Invitrogen), and examined for fluorescence. Images were acquired with a Zeiss LSM 780 confocal.

### Flow cytometric analysis of infected cells

RS cell monolayers were infected with 0.05 PFU/cell of recombinant GFP-McKrae or McKrae virus or were mock-infected for 18 h. Infected cells were harvested and stained with anti-gD antibody. Stained cells were washed 2× with FACS buffer (1× PBS with 0.1% sodium azide), resuspended in 4% paraformaldehyde, and analyzed in a BD LSR II flow cytometer using BD FacsDiva Software (BD Biosciences). Post-experiment data analysis was performed using FlowJo software (TreeStar).

### HSV-induced eye disease and angiogenesis

**T**he severity of corneal scarring/corneal disease lesions in mouse corneas was examined by slit lamp biomicroscopy on day 30 PI. The examination was conducted by an investigator blinded to the treatment regimen of the mice. Mice from each group were randomly screened without anesthesia for haziness. The scoring scale was as follows: 0, normal cornea; 1, mild haze; 2, moderate opacity; 3, severe corneal opacity but iris visible; 4, opaque and corneal ulcer; and 5, corneal rupture and necrotizing keratitis, as we previously described. The severity of angiogenesis on day 29 PI was recorded using a four-point scale in which a grade of 4 for a given quadrant of the circle represents centripetal growth of 1.5 mm toward the corneal center. Scores of the four eye quadrants were summed to derive the neovessel index (range, 0–16) for each eye at a given time point. The data were then plotted in prism to generate a graph, and nonparametric Mann–Whitney *t*-test with two tailed *P*-values was performed.

### Detecting GFP expression in corneas, whole eyes, and TG of infected mice

Three mice per group were ocularly infected with 2 × 10^5^ PFU/eye of GFP-McKrae virus. On days 2, 3 and 5 PI, the mice were euthanized, and their eyeballs were removed. Corneal tissue was separated, and the tissues were flattened with four partial cuts from the limbal to central cornea. Corneal tissue was mounted with Vectashield medium containing DAPI (Vector Laboratories, Burlingame, CA). Images were acquired as described above. For whole eye imaging, eyeballs were removed from the euthanized mice and stored in medium before processing. Eyeballs were kept on a dry paper towel to remove residual medium and transferred into OCT blocks. Eyeballs in OCT were immediately frozen on dry ice and transferred to −80°C. Eyes in the OCT blocks were thawed at −20°C before sectioning. For TG, mice were euthanized as described earlier. Individual TGs were separated from the brain and surgically removed. TG were stored in medium before processing. TG were transferred to OCT blocks and quickly frozen in dry ice. For sectioning, OCT blocks were removed from −80°C and sectioned as described earlier.

### TG RNA extraction, cDNA synthesis, and TaqMan RT-PCR

TG from individual mice were collected on day 30 PI, immersed in RNAlater RNA stabilization reagent, and stored at −80°C until processing. Qiazol RNA reagent (Qiagen) and BCP were used to extract RNA from individual TGs. Total RNA was extracted as we described previously (35, 40). Following RNA extraction, 1,000 ng of total RNA was reverse-transcribed using random hexamer primers and murine leukemia virus reverse transcriptase from the High Capacity cDNA Reverse Transcription Kit (Applied Biosystems, Foster City, CA), according to the manufacturer’s recommendations. Primer probe sets consisted of two unlabeled PCR primers and the FAM^TM^ dye-labeled TaqMan MGB probe formulated into a single mixture. The following assays were used in this study: 1) CD4 (ABI Mm00442754_m1, amplicon length = 72 bp); 2) CD8α (ABI Mm01182108_m1, amplicon length = 67 bp); 3) IFN-γ (Mm00801778_m1, amplicon length = 101 bp); 4) IFN-α2A (Mm00833961_s1, amplicon length = 158 bp); 5) IFN-β (Mm00439552_s1, amplicon length = 69 bp); 6) CD80 (MM00711660_m1, amplicon length  =  117 bp); 7) CD86 (Mm00444540_m1, amplicon length = 91 bp); 8) CD28 (Mm01253994_m1, amplicon length = 98 bp); 16) PD-L1 (Mm03048248_m1, amplicon length = 73 bp); 9) CTLA4 (Mm00486849_m1, amplicon length = 71 bp); 10) PD-1 (programmed death 1; ABI Mm00435532_m1; amplicon length = 65 bp); 11) TGFβ (Mm01178820_m1 – amplicon length = 59 bp); and 12) FoxP3 (Mm00475164_m1 – amplicon length = 80 bp). In all experiments, glyceraldehyde-3-phosphate dehydrogenase (GAPDH, Mm99999915_g1, Amplicon size 107 bp) was used to normalize transcripts. Expression levels of transcripts in naïve mice TG was used as a baseline control to estimate the relative expression of each transcript in TG of latently infected mice. A custom-made primer and probe set was used for latency-associated transcript (LAT) as follows: Forward- 5′-GGGTGGGCTCGTGTTA’AG-3′; Reverse’-, 5′-GGACGGGTAAGTAACAGAGTCT’TA-3′; and Probe- 5′-FAM-ACACCAGCCCGTTC’TT-3′, Amplicon length = 81 bp. For GFP, a custom TaqMan Gene Expression Assay, Catalog number: 4331348, Assay ID: AP9HURV. Transcripts in latent TG were evaluated using commercially available TaqMan Gene Expression Assays (Applied Biosystems, Foster City, CA, USA) with optimized primer and probe concentrations. The 2^−ΔΔ*CT*^ method was used to calculate fold change in gene expression relative to expression in uninfected controls.

### IHC staining

Eyes from GFP-McKrae- and WT-McKrae-infected mice were harvested on days 3, 5, and 30, frozen in OCT compound, and stored at −80°C until further processing. Uninfected mice were used as control. Frozen tissue was sectioned using a Cryostar NX70 (Thermo Scientific, Rockford, IL). Tissue sections were fixed with 4% paraformaldehyde, washed with 1× PBS, and then permeabilized with 0.1% Triton X-100 in PBS. The slides were blocked using blocking solution (2% BSA +2% goat serum +2% milk in 0.1% PBST) for 1 h at 25°C, then incubated with rat anti-GFP (Abcam- ab252881; 1:100), rabbit anti-gD (ThermoFisher- PA1-30233; 1:50) in blocking buffer at 4°C overnight. Slides were washed thrice with 1× PBST and incubated with goat anti-rat AF594 (ThermoFisher- A-11007; 1:100) and goat anti-rabbit AF647 (ThermoFisher- A-21245; 1:100) for 2 h at 25°C. Slides were then washed thrice with 1× PBST and developed using a Vector VIP substrate kit according to company instructions (Vector Laboratories, Burlingame, CA. Cat# SK-4600). Slides were prepared with Prolong Gold antifade reagent with DAPI (Invitrogen- P36931) mounting medium, and image acquisition and data analysis were performed using a Leica Leica Stellaris 8-STED Super-resolution Confocal Microscope (Leica Microsystems, Buffalo Grove, IL). Images were analyzed using LAS X Office 1.4.4 software. Whole TG images were acquired on a Zeiss AxioscanZ1 fluorescent microscope at 20× magnification and processed using ZEN lite (version: Zen 3.8) software.

### Imaging mass cytometry staining and data acquisition

#### Antibody validation

Antibodies used in this study (Table S1) were stored in 0.5 mg/mL aliquots at 4°C. To reduce channel overlap, which is minimal for the IMC platform and predominantly occurs between adjacent channels, we avoided placing low-intensity markers next to high-intensity markers during the panel design. Conjugation of antibodies with metal tags was performed following the manufacturer’s instructions. The antibody panel was titrated to identify the optimal titer for each antibody.

#### Tissue staining

Frozen mouse cornea samples were sectioned at 7 µm on a super frost plus slide and stored at −80°C. Before IMC staining, slides were air dried for 2 h, fixed in cold acetone for 10 min, then air dried for 30 min. A Pap pen was used to make a boundary around the sample and slides were soaked in 1× PBS for 5 min followed by soaking in 3% BSA at RT for 45 min. After discarding the BSA, a cocktail of meta-antibody conjugates to each antibody was applied to the sample and incubated overnight at 4°C in a humid chamber. After incubation, sections were washed twice for 8 min in a container with 0.1% Triton in 1× PBS and 1× PBS wash buffer, respectively. The slides were incubated with 1:500 dilution of Intercalator-Ir in 1× PBS for 40 min at RT. The slides were washed for 4 min with Milli-Q water at RT and air dried at RT.

#### IMC data acquisition

Slides were processed for ablation using a Hyperion imaging system (Standard Biotools) connected to a Helios mass cytometer (Standard Biotools) according to the manufacturer’s instructions. Resulting ionized isotope element plumes were captured by a mass cytometer to provide the number and type of metal isotopes per pixel (1 pixel = 1 µM × 1 µM). Acquired images were processed using dedicated imaging analysis software. Cell segmentation and feature extraction were performed to identify individual cells and quantify marker expression so that the spatial distribution of different cell types could be determined within the sample. Marker expression patterns of different sample groups were compared by statistical analysis. To ensure performance stability, the mass cytometer machine was calibrated daily with a tuning slide spiked with five metal elements (Fluidigm), and tissue slides were laser-ablated at a resolution of 1 mm (ablation energy: 2 dB) and a frequency of 200 Hz. A total of six (uninfected), seven (day 3) and ten (day 5) regions-of-interest (ROIs) were acquired and stored as individual MCD and.txt files. A selected areas (1–2 mm^2^) in each section was ablated by laser at a resolution of approximately 1 µm with a frequency of 200 Hz and an estimated acquisition time of 1 mm^2^ per hour. Selected areas for ablation were larger than the actual area of interest to account for loss of overlapping areas due to cumulative rotation of sections. All data were collected using commercial Fluidigm CyTOF software v.01.

#### Data structure and IMC analysis

Ablation sessions from the Fluidigm Hyperion imaging system produce MCD files that contain individual TXT ROI files for each acquisition. Due to the circular peripheral shape of corneas and optimization of ablation time, images of each cornea were obtained in rectangular tiles along the tissue and later computationally stitched (recombined) to reconstruct a single ROI per cornea by maximizing correlation versus offset of nuclear and other markers along the stitching edges of adjacent image tiles. Subsequent analysis included identifying cells via segmentation, obtaining expression, normalization, cell phenotyping, identifying spatial interactions, and quantifying phenotypes and functional expression across samples.

#### IMC pipeline steps

Cells were segmented using the DeepCell Mesmer model (68). Segmentation requires both a nuclear and membrane channel, which were obtained via Iridium-191/193 intercalator staining and a pseudo-membrane channel based on edge detection of nuclei as we did not have a single ubiquitously expressed membrane marker. In the resulting cell masks, pixels were labeled by the index of the cell they belong to. Cell expression was determined by averaging the pixel values of each marker over each cell. Expression was normalized by log-scaling raw expression and soft-clipping the upper range by applying a hyperbolic tangent with the upper “elbow” of the expression quantile plot across the data set as a cofactor for each individual marker. This was done to attenuate the impact of expression outliers during subsequent clustering and phenotyping by applying community detection *via* Leiden clustering on phenotype-associated lineage markers within each sample (69), following the same logic as in the phenograph method (70). In addition to clustering cells based on expression, we also clustered on spatial location with high resolution – resulting in 10 to 20 cells per spatial cluster (i.e., local niche) – using Leiden again with a nearest-neighbor setting of 5. Having both data-driven expression clusters (pseudo-phenotypes) and spatial clusters, we could infer phenotypic proportions within each local niche and represent phenotypic spatial interactions by calculating pairwise correlations of phenotype enrichment across sample niches for all phenotype clusters. We show clustered heatmaps indicating phenotypic spatial interactions based on these correlations.

#### IMC staining and analysis

Staining was standardized on a small region of mouse corneas to visualize GFP-McKrae on day 3 PI, and single GFP-positive ROI was assigned for analysis that detected 25 different protein markers including GFP. Live and dead cells were identified by expression of Ki67 and cleaved Caspase 3, respectively. Markers that did not show clear staining, including CD19, CD44, Tbet, CD138, MMP9, GATA3, and Granzyme B, were removed from the analysis, leaving the detectable markers CD11b, CD11c, CD45, CD163, CD3, CD4, F4-80, CD8a, CD68, Ly-6G, iNOS, FoxP3, E-cadherin, and GFP. Each antibody resulted in a single image per sample, and collectively was used to construct a multi-image stack in MCD format. By applying hierarchical and Leiden clustering to IMC single-cell data as described above, we obtained data-driven immune phenotyping (Fig. S1A). After cell segmentation, feature extraction, and normalization, cell populations were identified using iterative PhenoGraph clustering. Final matrix data were converted to .FCS files within the MATLAB pipeline for clustering and heatmap creation. Several immune cell markers were identified as expected (Fig. S1B). Cells with GFP-positive regions were spatially located on the cornea (Fig. S1C). Individual markers or in combination identity immune cells as described in Table S1. Notably, not all immune cells were CD45 positive, a discrepancy previously observed by others (71–73). Neighborhood analysis was used to identify closely situated clusters (Fig. S1D), and GFP distribution was used to identify GFP-positive cells (Fig. S1E). Individual clusters were analyzed to determine the fraction (Fig. S1F) and number of cells (Fig. S1G).

### Statistical analysis

For all statistical tests, *P* ≤ 0.05 was considered statistically significant and indicated by a single asterisk (*), and *P* ≤ 0.001 are indicated by double asterisks (**). A two-tailed Student *t* test with unequal variances was used to compare differences between two experimental groups. A one-way analysis of variance (ANOVA) was used to compare differences among three or more experimental groups. All experiments were repeated at least thrice to ensure accuracy.

## SUPPLEMENTAL MATERIAL

Fig. S1 mbio.01454-24-s0001.tiff Optimization of IMC protocol in cornea of GFP-McKrae-infected mice on day 3 PI.

Fig. S2 mbio.01454-24-s0002.tiff Spatial analysis of corneas infected with GFP-McKrae virus.

Fig. S3 mbio.01454-24-s0003.tif Detection of GFP and gD in corneas of GFP-McKrae- or McKrae-infected mice on day 5 PI.

Fig. S4 mbio.01454-24-s0004.tif Detection of GFP, gD, or HSV-1 antigens on day 5 PI in TG of GFP-McKrae- or McKrae-infected mice.

Titles mbio.01454-24-s0005.pdf Titles of Table S1 and Fig. S1 to S3.

Table S1 mbio.01454-24-s0006.pdf List of antibodies used for IMC.

ASM does not own the copyrights to Supplemental Material that may be linked to, or accessed through, an article. The authors have granted ASM a non-exclusive, world-wide license to publish the Supplemental Material files. Please contact the corresponding author directly for reuse.

## References

[1] Oh JO, Kimura SJ, Ostler HB, Dawson CR, Smolin G. 1976. Oculogenital transmission of type 2 herpes simplex virus in adults. Surv Ophthalmol 21:106–109. doi:10.1016/0039-6257(76)90087-4 185732

[2] Pavan-Langston D. 1983. Ocular viral infections. Med Clin North Am 67:973–990. doi:10.1016/s0025-7125(16)31162-2 6312219

[3] Corey L. 1994. The current trend in genital herpes. Progress in prevention. Sex Transm Dis 21:S38–44. 8042114

[4] Liesegang TJ, Melton LJ, Daly PJ, Ilstrup DM. 1989. Epidemiology of ocular herpes simplex. Incidence in Rochester, Minn, 1950 through 1982. Arch Ophthalmol 107:1155–1159. doi:10.1001/archopht.1989.01070020221029 2787981

[5] Barron BA, Gee L, Hauck WW, Kurinij N, Dawson CR, Jones DB, Wilhelmus KR, Kaufman HE, Sugar J, Hyndiuk RA. 1994. Herpetic eye disease study. A controlled trial of oral acyclovir for herpes simplex stromal keratitis. Ophthalmology 101:1871–1882. doi:10.1016/s0161-6420(13)31155-5 7997323

[6] Wilhelmus KR, Dawson CR, Barron BA, Bacchetti P, Gee L, Jones DB, Kaufman HE, Sugar J, Hyndiuk RA, Laibson PR, Stulting RD, Asbell PA. 1996. Risk factors for herpes simplex virus epithelial keratitis recurring during treatment of stromal keratitis or iridocyclitis. Herpetic eye disease study group. Br J Ophthalmol 80:969–972. doi:10.1136/bjo.80.11.969 8976723 PMC505673

[7] Liesegang TJ. 1999. Classification of herpes simplex virus keratitis and anterior uveitis. Cornea 18:127–143. doi:10.1097/00003226-199903000-00001 10090358

[8] Liesegang TJ. 2001. Herpes simplex virus epidemiology and ocular importance. Cornea 20:1–13. doi:10.1097/00003226-200101000-00001 11188989

[9] Hill TJ. 1987. Ocular pathogenicity of herpes simplex virus. Curr Eye Res 6:1–7. doi:10.3109/02713688709020060 3030632

[10] Diefenbach RJ, Miranda-Saksena M, Douglas MW, Cunningham AL. 2008. Transport and egress of herpes simplex virus in neurons. Rev Med Virol 18:35–51. doi:10.1002/rmv.560 17992661

[11] Feierbach B, Bisher M, Goodhouse J, Enquist LW. 2007. In vitro analysis of transneuronal spread of an alphaherpesvirus infection in peripheral nervous system neurons. J Virol 81:6846–6857. doi:10.1128/JVI.00069-07 17459934 PMC1933274

[12] Stevens JG. 1989. Human herpesviruses: a consideration of the latent state. Microbiol Rev 53:318–332. doi:10.1128/mr.53.3.318-332.1989 2552271 PMC372739

[13] Wechsler SL, Nesburn AB, Watson R, Slanina S, Ghiasi H. 1988. Fine mapping of the major latency-related RNA of herpes simplex virus type 1 in humans. J Gen Virol 69 (Pt 12):3101–3106. doi:10.1099/0022-1317-69-12-3101 2848928

[14] Fraser NW, Valyi-Nagy T. 1993. Viral, neuronal and immune factors which may influence herpes simplex virus (HSV) latency and reactivation. Microb Pathog 15:83–91. doi:10.1006/mpat.1993.1059 8255209

[15] Streilein JW. 2003. Ocular immune privilege: the eye takes a dim but practical view of immunity and inflammation. J Leukoc Biol 74:179–185. doi:10.1189/jlb.1102574 12885934

[16] Green DR, Ferguson TA. 2001. The role of Fas ligand in immune privilege. Nat Rev Mol Cell Biol 2:917–924. doi:10.1038/35103104 11733771

[17] Cursiefen C, Chen L, Dana MR, Streilein JW. 2003. Corneal lymphangiogenesis: evidence, mechanisms, and implications for corneal transplant immunology. Cornea 22:273–281. doi:10.1097/00003226-200304000-00021 12658100

[18] Iwamoto T, Smelser GK. 1965. Electron microscope studies on the mast cells and blood and lymphatic capillaries of the human corneal limbus. Invest Ophthalmol 4:815–834. 5831990

[19] Planck SR, Rich LF, Ansel JC, Huang XN, Rosenbaum JT. 1997. Trauma and alkali burns induce distinct patterns of cytokine gene expression in the rat cornea. Ocul Immunol Inflamm 5:95–100. doi:10.3109/09273949709085057 9234373

[20] Rosenbaum JT, Planck ST, Huang XN, Rich L, Ansel JC. 1995. Detection of mRNA for the cytokines, interleukin-1 alpha and interleukin-8, in corneas from patients with pseudophakic bullous keratopathy. Invest Ophthalmol Vis Sci 36:2151–2155. 7657553

[21] Kuffova L, Lumsden L, Forrester JV, Filipec M. 1999. Cell subpopulations in failed human corneal grafts. Br J Ophthalmol 83:1364–1369. doi:10.1136/bjo.83.12.1364 10574815 PMC1722894

[22] Hazlett LD, McClellan SA, Rudner XL, Barrett RP. 2002. The role of Langerhans cells in Pseudomonas aeruginosa infection. Invest Ophthalmol Vis Sci 43:189–197. 11773031

[23] Hazlett LD, McClellan S, Kwon B, Barrett R. 2000. Increased severity of Pseudomonas aeruginosa corneal infection in strains of mice designated as Th1 versus Th2 responsive. Invest Ophthalmol Vis Sci 41:805–810. 10711697

[24] Ghiasi H, Wechsler SL, Cai S, Nesburn AB, Hofman FM. 1998. The role of neutralizing antibody and T-helper subtypes in protection and pathogenesis of vaccinated mice following ocular HSV-1 challenge. Immunology 95:352–359. doi:10.1046/j.1365-2567.1998.00602.x 9824497 PMC1364400

[25] Ghiasi H, Wechsler SL, Kaiwar R, Nesburn AB, Hofman FM. 1995. Local expression of tumor necrosis factor alpha and interleukin-2 correlates with protection against corneal scarring after ocular challenge of vaccinated mice with herpes simplex virus type 1. J Virol 69:334–340. doi:10.1128/jvi.69.1.334-340.1995 7983727 PMC188580

[26] Lee DH, Jaggi U, Ghiasi H. 2019. CCR2^+^ migratory macrophages with M1 status are the early-responders in the cornea of HSV-1 infected mice. PLoS One 14:e0215727. doi:10.1371/journal.pone.0215727 30998796 PMC6472814

[27] Balliet JW, Kushnir AS, Schaffer PA. 2007. Construction and characterization of a herpes simplex virus type I recombinant expressing green fluorescent protein: acute phase replication and reactivation in mice. Virology (Auckl) 361:372–383. doi:10.1016/j.virol.2006.11.022 PMC1975764 17207829

[28] Elliott G, O’Hare P. 1999. Live-cell analysis of a green fluorescent protein-tagged herpes simplex virus infection. J Virol 73:4110–4119. doi:10.1128/JVI.73.5.4110-4119.1999 10196307 PMC104190

[29] Ramachandran S, Knickelbein JE, Ferko C, Hendricks RL, Kinchington PR. 2008. Development and pathogenic evaluation of recombinant herpes simplex virus type 1 expressing two fluorescent reporter genes from different lytic promoters. Virology (Auckl) 378:254–264. doi:10.1016/j.virol.2008.05.034 PMC2613845 18619637

[30] Uyar O, Plante PL, Piret J, Venable MC, Carbonneau J, Corbeil J, Boivin G. 2021. A novel bioluminescent herpes simplex virus 1 for in vivo monitoring of herpes simplex encephalitis. Sci Rep 11:18688. doi:10.1038/s41598-021-98047-z 34548521 PMC8455621

[31] Snyder A, Bruun B, Browne HM, Johnson DC. 2007. A herpes simplex virus gD-YFP fusion glycoprotein is transported separately from viral capsids in neuronal axons. J Virol 81:8337–8340. doi:10.1128/JVI.00520-07 17522199 PMC1951318

[32] Kapoor D, Sharma P, Shukla D. 2024. Emerging drugs for the treatment of herpetic keratitis. Expert Opin Emerg Drugs 29:113–126. doi:10.1080/14728214.2024.2339899 38603466 PMC12558075

[33] McGeoch DJ, Dalrymple MA, Davison AJ, Dolan A, Frame MC, McNab D, Perry LJ, Scott JE, Taylor P. 1988. The complete DNA sequence of the long unique region in the genome of herpes simplex virus type 1. J Gen Virol 69:1531–1574. doi:10.1099/0022-1317-69-7-1531 2839594

[34] Watson G, Xu W, Reed A, Babra B, Putman T, Wick E, Wechsler SL, Rohrmann GF, Jin L. 2012. Sequence and comparative analysis of the genome of HSV-1 strain McKrae. Virology (Auckl) 433:528–537. doi:10.1016/j.virol.2012.08.043 23021301

[35] Mott KR, Perng GC, Osorio Y, Kousoulas KG, Ghiasi H. 2007. A recombinant herpes simplex virus type 1 expressing two additional copies of gK is more pathogenic than wild-type virus in two different strains of mice. J Virol 81:12962–12972. doi:10.1128/JVI.01442-07 17898051 PMC2169076

[36] Kuett L, Catena R, Özcan A, Plüss A, Cancer Grand Challenges IMAXT Consortium, Schraml P, Moch H, de Souza N, Bodenmiller B. 2022. Three-dimensional imaging mass cytometry for highly multiplexed molecular and cellular mapping of tissues and the tumor microenvironment. Nat Cancer 3:122–133. doi:10.1038/s43018-021-00301-w 35121992 PMC7613779

[37] Giesen C, Wang HAO, Schapiro D, Zivanovic N, Jacobs A, Hattendorf B, Schüffler PJ, Grolimund D, Buhmann JM, Brandt S, Varga Z, Wild PJ, Günther D, Bodenmiller B. 2014. Highly multiplexed imaging of tumor tissues with subcellular resolution by mass cytometry. Nat Methods 11:417–422. doi:10.1038/nmeth.2869 24584193

[38] Rendeiro AF, Ravichandran H, Bram Y, Chandar V, Kim J, Meydan C, Park J, Foox J, Hether T, Warren S, Kim Y, Reeves J, Salvatore S, Mason CE, Swanson EC, Borczuk AC, Elemento O, Schwartz RE. 2021. The spatial landscape of lung pathology during COVID-19 progression. Nature New Biol 593:564–569. doi:10.1038/s41586-021-03475-6 PMC8204801 33780969

[39] Pauker VI, Bertzbach LD, Hohmann A, Kheimar A, Teifke JP, Mettenleiter TC, Karger A, Kaufer BB. 2019. Imaging mass spectrometry and proteome analysis of Marek’s disease virus-induced tumors. mSphere 4:e00569-18. doi:10.1128/mSphere.00569-18 30651403 PMC6336081

[40] Mott KR, Osorio Y, Brown DJ, Morishige N, Wahlert A, Jester JV, Ghiasi H. 2007. The corneas of naive mice contain both CD4^+^ and CD8^+^ T cells. Mol Vis 13:1802–1812. 17960132

[41] Wang S, Ljubimov AV, Jin L, Pfeffer K, Kronenberg M, Ghiasi H. 2018. Herpes simplex virus 1 latency and the kinetics of reactivation are regulated by a complex network of interactions between the herpesvirus entry mediator, its ligands (gD, BTLA, LIGHT, and CD160), and the latency-associated transcript. J Virol 92:e01451-18. doi:10.1128/JVI.01451-18 PMC6258941 30282707

[42] Allen SJ, Rhode-Kurnow A, Mott KR, Jiang X, Carpenter D, Rodriguez-Barbosa JI, Jones C, Wechsler SL, Ware CF, Ghiasi H. 2014. Interactions between herpesvirus entry mediator (TNFRSF14) and latency-associated transcript during herpes simplex virus 1 latency. J Virol 88:1961–1971. doi:10.1128/JVI.02467-13 24307582 PMC3911542

[43] Ghiasi H, Nesburn AB, Kaiwar R, Wechsler SL. 1991. Immunoselection of recombinant baculoviruses expressing high levels of biologically active herpes simplex virus type 1 glycoprotein D. Arch Virol 121:163–178. doi:10.1007/BF01316752 1662037

[44] Hogue I, Bosse J, Engel E, Scherer J, Hu J-R, Del Rio T, Enquist L. 2015. Fluorescent protein approaches in alpha herpesvirus research. Viruses 7:5933–5961. doi:10.3390/v7112915 26610544 PMC4664988

[45] Aboody-Guterman KS, Pechan PA, Rainov NG, Sena-Esteves M, Jacobs A, Snyder EY, Wild P, Schraner E, Tobler K, Breakefield XO, Fraefel C. 1997. Green fluorescent protein as a reporter for retrovirus and helper virus-free HSV-1 amplicon vector-mediated gene transfer into neural cells in culture and in vivo. Neuroreport 8:3801–3808. doi:10.1097/00001756-199712010-00029 9427374

[46] Feltrin C, Oliveira Simões CM, Marques Sincero TC. 2022. Development of a cell-based reporter assay for detection of human alphaherpesviruses. Mol Cell Probes 62:101806. doi:10.1016/j.mcp.2022.101806 35257855

[47] David AT, Saied A, Charles A, Subramanian R, Chouljenko VN, Kousoulas KG. 2012. A herpes simplex virus 1 (McKrae) mutant lacking the glycoprotein K gene is unable to infect via neuronal axons and egress from neuronal cell bodies. MBio 3:e00144-12. doi:10.1128/mBio.00144-12 22829677 PMC3413403

[48] Proença JT, Nelson D, Nicoll MP, Connor V, Efstathiou S. 2016. Analyses of herpes simplex virus type 1 latency and reactivation at the single cell level using fluorescent reporter mice. J Gen Virol 97:767–777. doi:10.1099/jgv.0.000380 26694770 PMC5381395

[49] Scherer KM, Manton JD, Soh TK, Mascheroni L, Connor V, Crump CM, Kaminski CF. 2021. A fluorescent reporter system enables spatiotemporal analysis of host cell modification during herpes simplex virus-1 replication. J Biol Chem 296:100236. doi:10.1074/jbc.RA120.016571 33380421 PMC7948757

[50] Elliott G, O’Hare P. 1999. Intercellular trafficking of VP22-GFP fusion proteins. Gene Ther 6:149–151. doi:10.1038/sj.gt.3300850 10341888

[51] Nicoll MP, Hann W, Shivkumar M, Harman LER, Connor V, Coleman HM, Proença JT, Efstathiou S. 2016. The HSV-1 latency-associated transcript functions to repress latent phase lytic gene expression and suppress virus reactivation from latently infected neurons. PLoS Pathog 12:e1005539. doi:10.1371/journal.ppat.1005539 27055281 PMC4824392

[52] Liu J, Xue Y, Dong D, Xiao C, Lin C, Wang H, Song F, Fu T, Wang Z, Chen J, Pan H, Li Y, Cai D, Li Z. 2017. CCR2− and CCR2^+^ corneal macrophages exhibit distinct characteristics and balance inflammatory responses after epithelial abrasion. Mucosal Immunol 10:1145–1159. doi:10.1038/mi.2016.139 28120849 PMC5562841

[53] Caetano AJ, Redhead Y, Karim F, Dhami P, Kannambath S, Nuamah R, Volponi AA, Nibali L, Booth V, D’Agostino EM, Sharpe PT. 2023. Spatially resolved transcriptomics reveals pro-inflammatory fibroblast involved in lymphocyte recruitment through CXCL8 and CXCL10. Elife 12:e81525. doi:10.7554/eLife.81525 36648332 PMC9897724

[54] Biswas PS, Rouse BT. 2005. Early events in HSV keratitis--setting the stage for a blinding disease. Microbes Infect 7:799–810. doi:10.1016/j.micinf.2005.03.003 15857807

[55] Feldman LT, Ellison AR, Voytek CC, Yang L, Krause P, Margolis TP. 2002. Spontaneous molecular reactivation of herpes simplex virus type 1 latency in mice. Proc Natl Acad Sci U S A 99:978–983. doi:10.1073/pnas.022301899 11773630 PMC117416

[56] Kramer MF, Coen DM. 1995. Quantification of transcripts from the ICP4 and thymidine kinase genes in mouse ganglia latently infected with herpes simplex virus. J Virol 69:1389–1399. doi:10.1128/JVI.69.3.1389-1399.1995 7853471 PMC188725

[57] Margolis TP, Elfman FL, Leib D, Pakpour N, Apakupakul K, Imai Y, Voytek C. 2007. Spontaneous reactivation of herpes simplex virus type 1 in latently infected murine sensory ganglia. J Virol 81:11069–11074. doi:10.1128/JVI.00243-07 17686862 PMC2045564

[58] Derfuss T, Segerer S, Herberger S, Sinicina I, Hüfner K, Ebelt K, Knaus H-G, Steiner I, Meinl E, Dornmair K, Arbusow V, Strupp M, Brandt T, Theil D. 2007. Presence of HSV-1 immediate early genes and clonally expanded T-cells with a memory effector phenotype in human trigeminal ganglia. Brain Pathol 17:389–398. doi:10.1111/j.1750-3639.2007.00088.x 17784877 PMC8095593

[59] Held K, Junker A, Dornmair K, Meinl E, Sinicina I, Brandt T, Theil D, Derfuss T. 2011. Expression of herpes simplex virus 1-encoded microRNAs in human trigeminal ganglia and their relation to local T-cell infiltrates. J Virol 85:9680–9685. doi:10.1128/JVI.00874-11 21795359 PMC3196425

[60] Desai P, Person S. 1998. Incorporation of the green fluorescent protein into the herpes simplex virus type 1 capsid. J Virol 72:7563–7568. doi:10.1128/JVI.72.9.7563-7568.1998 9696854 PMC110002

[61] Ghiasi H, Osorio Y, Hedvat Y, Perng GC, Nesburn AB, Wechsler SL. 2002. Infection of BALB/c mice with a herpes simplex virus type 1 recombinant virus expressing IFN-γ driven by the LAT promoter. Virology (Auckl) 302:144–154. doi:10.1006/viro.2002.1609 12429523

[62] Ghiasi H, Osorio Y, Perng GC, Nesburn AB, Wechsler SL. 2002. Overexpression of interleukin-2 by a recombinant herpes simplex virus type 1 attenuates pathogenicity and enhances antiviral immunity. J Virol 76:9069–9078. doi:10.1128/jvi.76.18.9069-9078.2002 12186890 PMC136420

[63] Ghiasi H, Kaiwar R, Nesburn AB, Slanina S, Wechsler SL. 1994. Expression of seven herpes simplex virus type 1 glycoproteins (gB, gC, gD, gE, gG, gH, and gI): comparative protection against lethal challenge in mice. J Virol 68:2118–2126. doi:10.1128/JVI.68.4.2118-2126.1994 8138996 PMC236686

[64] Ghiasi H, Kaiwar R, Nesburn AB, Wechsler SL. 1992. Expression of herpes simplex virus type 1 glycoprotein B in insect cells. Initial analysis of its biochemical and immunological properties. Virus Res 22:25–39. doi:10.1016/0168-1702(92)90087-p 1311136

[65] Ghiasi H, Kaiwar R, Nesburn AB, Wechsler SL. 1992. Baculovirus expressed herpes simplex virus type 1 glycoprotein C protects mice from lethal HSV-1 infection. Antiviral Res 18:291–302. doi:10.1016/0166-3542(92)90062-a 1416910

[66] Ghiasi H, Bahri S, Nesburn AB, Wechsler SL. 1995. Protection against herpes simplex virus-induced eye disease after vaccination with seven individually expressed herpes simplex virus 1 glycoproteins. Invest Ophthalmol Vis Sci 36:1352–1360. 7775113

[67] Mott KR, Ghiasi H. 2008. Role of dendritic cells in enhancement of herpes simplex virus type 1 latency and reactivation in vaccinated mice. Clin Vaccine Immunol 15:1859–1867. doi:10.1128/CVI.00318-08 18971304 PMC2593179

[68] Greenwald NF, Miller G, Moen E, Kong A, Kagel A, Dougherty T, Fullaway CC, McIntosh BJ, Leow KX, Schwartz MS, et al.. 2022. Whole-cell segmentation of tissue images with human-level performance using large-scale data annotation and deep learning. Nat Biotechnol 40:555–565. doi:10.1038/s41587-021-01094-0 34795433 PMC9010346

[69] Traag VA, Waltman L, van Eck NJ. 2019. From louvain to leiden: guaranteeing well-connected communities. Sci Rep 9:5233. doi:10.1038/s41598-019-41695-z 30914743 PMC6435756

[70] Levine JH, Simonds EF, Bendall SC, Davis KL, Amir ED, Tadmor MD, Litvin O, Fienberg HG, Jager A, Zunder ER, Finck R, Gedman AL, Radtke I, Downing JR, Pe’er D, Nolan GP. 2015. Data-driven phenotypic dissection of AML reveals progenitor-like cells that correlate with prognosis. Cell 162:184–197. doi:10.1016/j.cell.2015.05.047 26095251 PMC4508757

[71] Jackson HW, Fischer JR, Zanotelli VRT, Ali HR, Mechera R, Soysal SD, Moch H, Muenst S, Varga Z, Weber WP, Bodenmiller B. 2020. The single-cell pathology landscape of breast cancer. Nature New Biol 578:615–620. doi:10.1038/s41586-019-1876-x 31959985

[72] Ung N, Goldbeck C, Man C, Hoeflich J, Sun R, Barbetta A, Matasci N, Katz J, Lee JSH, Chopra S, Asgharzadeh S, Warren M, Sher L, Kohli R, Akbari O, Genyk Y, Emamaullee J. 2022. Adaptation of imaging mass cytometry to explore the single cell alloimmune landscape of liver transplant rejection. Front Immunol 13:831103. doi:10.3389/fimmu.2022.831103 35432320 PMC9009043

[73] Xie S, Zhang XY, Shan XF, Yau V, Zhang JY, Wang W, Yan YP, Cai ZG. 2021. Hyperion image analysis depicts a preliminary landscape of tumor immune microenvironment in OSCC with lymph node metastasis. J Immunol Res 2021:9975423. doi:10.1155/2021/9975423 34239944 PMC8238606

