# Supplementary figures and images for: A novel GFP-based strategy to quantitate cellular spatial associations in HSV-1 viral pathogenesis
Source: mBio. 2024 Sep 9;15(10):e01454-24. doi: 10.1128/mbio.01454-24 (PMC11481894; doi:10.1128/mbio.01454-24)

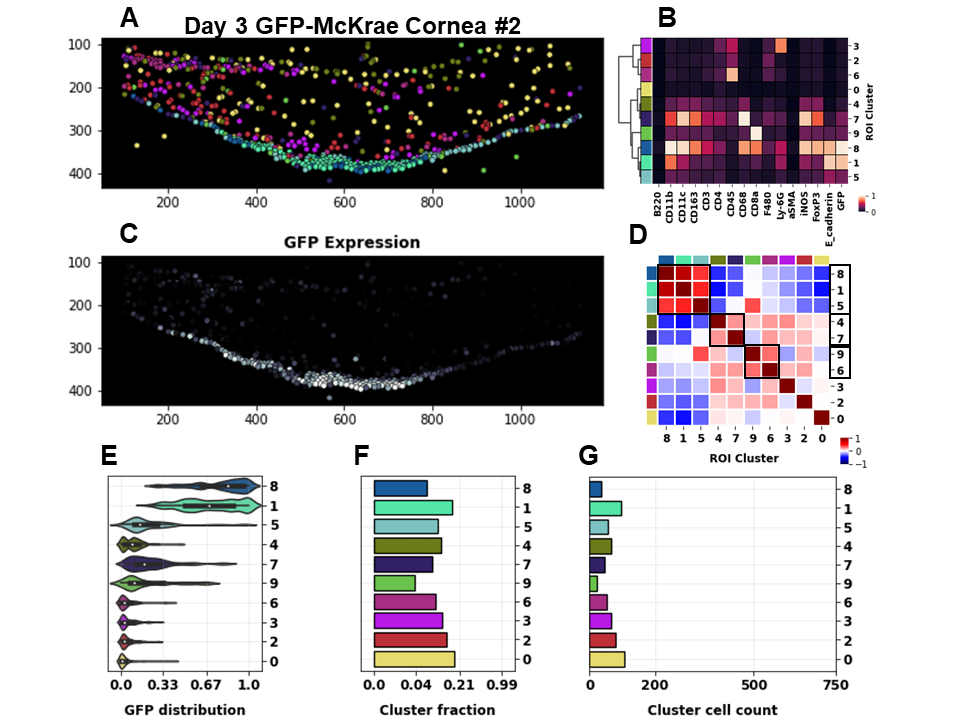

Supplement: Fig. S1 — Optimization of IMC protocol in cornea of GFP-McKrae-infected mice on day 3 PI. [file mbio.01454-24-s0001.tiff]

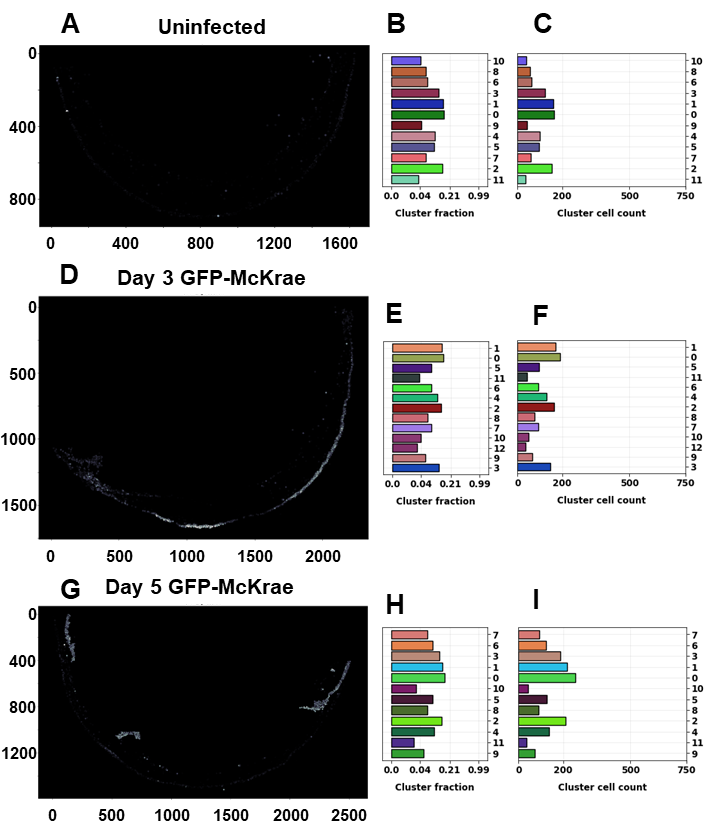

Supplement: Fig. S2 — Spatial analysis of corneas infected with GFP-McKrae virus. [file mbio.01454-24-s0002.tiff]

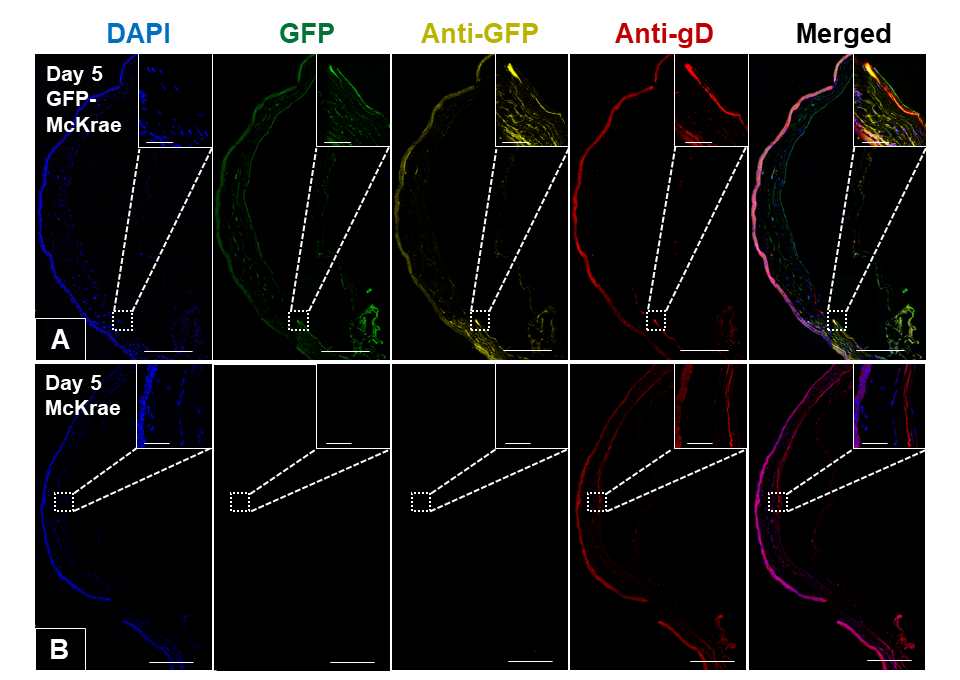

Supplement: Fig. S3 — Detection of GFP and gD in corneas of GFP-McKrae- or McKrae-infected mice on day 5 PI. [file mbio.01454-24-s0003.tif]

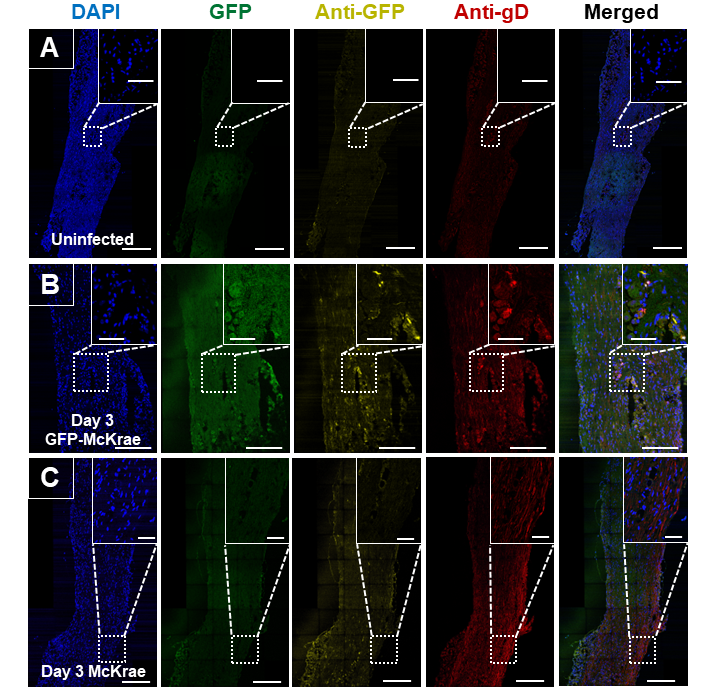

Supplement: Fig. S4 — Detection of GFP, gD, or HSV-1 antigens on day 5 PI in TG of GFP-McKrae- or McKrae-infected mice. [file mbio.01454-24-s0004.tif]
