# Supplementary material for: A novel GFP-based strategy to quantitate cellular spatial associations in HSV-1 viral pathogenesis
Source: mBio. 2024 Sep 9;15(10):e01454-24. doi: 10.1128/mbio.01454-24 (PMC11481894; doi:10.1128/mbio.01454-24)
Supplement: Titles — of Table S1 and Fig. S1 to S3. [file mbio.01454-24-s0005.pdf]

Supplementary Legends

<u>Table S1</u>. List of antibodies used for IMC.

<u>Fig. S1</u>. <u>Optimization of IMC protocol in cornea of GFP-McKrae-infected mice on day 3 PI</u>. IMC staining and analysis was performed as described in Fig. 5 and Materials and Methods. Single region of interest (ROI) from a different sample was acquired and analyzed for spatial profiling of cells. (A) Single-cell map was generated using spatial data acquired from single ROI. (B) Heatmap analysis shows normalized expression of structural, immune cell markers, and viral protein (GFP) in assigned clusters of cells. (C) GFP expression shown in reconstructed cornea, and individual cell clusters. (D) Neighborhood analysis as heatmap shows cluster proximity with each other. (E) Violin plot of GFP distribution measured in individual clusters (0-8) ranging 0 to 1. (F) Individual clusters (0-8), plotted as bar graph on a log scale to show the fraction of individual cluster in every sample. (G) Single-cell counts of each cluster (0-8), shown as bar graph, ranging from 0 to 750.

<u>Fig. S2. Spatial analysis of corneas infected with GFP-McKrae virus</u>. The description of each panel is explained in Fig. 5, and Supplementary Fig. 1. GFP expression in reconstructed cornea (A), fraction (B), and cell counts (C) from each cluster present in each sample are shown for uninfected cornea. (Fig. S2D-F and S2G-I) Spatial analysis of corneas infected with GFP-McKrae on days 3 and 5 PI, respectively. Similar data points were collected and plotted as in Fig. S2A-C.

<u>Fig. S3. Detection of GFP and gD in corneas of GFP-McKrae or McKrae-infected mice on day 5 PI.</u> Infection, cornea staining, image acquisition. and image processing in mice were performed as described in Fig. 6. Representative confocal images show staining of GFP, anti-GFP, and anti-gD in <u>GFP-McKrae (A) and McKrae (B)</u> infected corneas on day 5 PI. Scalebars represent 250μM. Enlarged colocalized region insets shown on top right. Scalebar represents 50μM.

<u>Fig. S4. Detection of GFP, gD, or HSV-1 antigens on day 5 PI in TG of GFP-McKrae- or McKrae-infected mice</u>. Infection, tissue harvesting, staining and image analysis were performed as described in Fig.

7 and 8. (A) Detection of HSV-1 in TG infected with GFP-McKrae virus on day 5 PI. (B-C) Expression of GFP, anti-GFP, and anti-gD in GFP-McKrae (B) or McKrae (C) infected TG, on day 3 PI.
