# Supplementary material for: A novel GFP-based strategy to quantitate cellular spatial associations in HSV-1 viral pathogenesis
Source: mBio. 2024 Sep 9;15(10):e01454-24. doi: 10.1128/mbio.01454-24 (PMC11481894; doi:10.1128/mbio.01454-24)
Supplement: Table S1 — List of antibodies used for IMC. [file mbio.01454-24-s0006.pdf]

	STD panel		
**N**	**Metal**	**Antibody**	**Cell subsets**
1	113In	aSMA	myofibroblast
2	142Nd	CD11c	DC
3	145Nd	Tbet	TH1
4	147Sm	CD163	M2 macrophage
5	149Sm	CD11b	macrophage
6	150Nd	CD68	macrophage
7	151Eu	CD138	syndecan-1/PG/plasma cell
8	152Sm	MMP9	protease
9	153Eu	CD44	activation/migration
10	155Gd	FOXP3	Treg
11	156Gd	CD4	T-cell
12	158Gd	E-cadherin	epithelium
13	159TB	F480	macrophage
14	160Gd	GATA3	TH2
15	161Dy	B220	B-cell
16	162Dy	CD8a	T-cell
17	164Dy	Ly-6G	neutrophil
18	166Er	iNOS	M1 macrophage
19	167Er	Granzyme B	cytotoxic, serine protease
20	170Er	CD3	T-cell
21	172Yb	Caspase 3, cleaved	apoptosis
22	196Pt	Ki-67	proliferation
23	209Bi	Histone H3	nuclear
24	89Y	CD45	hematopoietic
25	165Ho	GFP	GFP-McKrae
26	191/193Ir		Nucleus
